# A randomized controlled trial of simulation training in teaching coronary angiographic views

**DOI:** 10.1186/s12909-022-03705-z

**Published:** 2022-08-26

**Authors:** Kwan S. Lee, Balaji Natarajan, Wei X. Wong, Wina Yousman, Stefan Koester, Iwan Nyotowidjojo, Justin Z. Lee, Karl B. Kern, Deepak Acharya, David Fortuin, Olivia Hung, Wolfram Voelker, Julia H. Indik

**Affiliations:** 1 Sarver Heart Center, University of Arizona, 1501 North Campbell Avenue, Tucson, AZ 85724 USA; 2 University of California Riverside School of Medicine, Riverside, CA USA; 3 Mayo Clinic Arizona, Phoenix, AZ USA; 4 University Medical Center Wuerzburg, Wuerzburg, Germany

**Keywords:** Simulation training, Clinical competence, Diagnostic angiography

## Abstract

**Introduction:**

Simulation technology has an established role in teaching technical skills to cardiology fellows, but its impact on teaching trainees to interpret coronary angiographic (CA) images has not been systematically studied. The aim of this randomized controlled study was to test whether structured simulation training, in addition to traditional methods would improve CA image interpretation skills in a heterogeneous group of medical trainees.

**Methods:**

We prospectively randomized a convenience sample of 105 subjects comprising of medical students (*N* = 20), residents (*N* = 68) and fellows (*N* = 17) from the University of Arizona. Subjects were randomized in a stratified fashion into a simulation training group which received simulation training in addition to didactic teaching (*n* = 53) and a control training group which received didactic teaching alone (*n* = 52). The change in pre and post-test score (delta score) was analyzed by a two-way ANOVA for education status and training arm.

**Results:**

Subjects improved in their post-test scores with a mean change of 4.6 ± 4.0 points. Subjects in the simulation training arm had a higher delta score compared to control (5.4 ± 4.2 versus 3.8 ± 3.7, *p* = 0.04), with greatest impact for residents (6.6 ± 4.0 versus 3.5 ± 3.4) with a *p* = 0.02 for interaction of training arm and education status.

**Conclusions:**

Simulation training complements traditional methods to improve CA interpretation skill, with greatest impact on residents. This highlights the importance of incorporating high-fidelity simulation training early in cardiovascular fellowship curricula.

**Supplementary Information:**

The online version contains supplementary material available at 10.1186/s12909-022-03705-z.

## Introduction

Clinical educators are constantly innovating to address the challenge of effectively teaching students with ever increasing time constraints, medical complexity, and performance evaluation expectations [1]. In recent years, technology-enhanced simulation training in health sciences has been established to have large effects on outcomes of knowledge, skills and behaviors with modest effects on patient related outcomes in comparison with traditional learning [2–4]. The American Council for Graduate Medical Education (ACGME) statement of mandatory fellow participation in training has therefore recommended using simulation as a condition of accreditation for general cardiovascular and all cardiovascular sub-specialties. However, the exact methodology, implementation and educational objectives for simulation remains undefined [5, 6].

In the cardiac catheterization laboratory, several small randomized and non-randomized trials have shown the superiority of global and technical performance scores encompassing multiple elements of performance relating to diagnostic coronary angiography [7, 8], percutaneous coronary intervention [9, 10] and trans-septal puncture [11]. Mentored simulation training may also have the potential to reduce procedural errors [7, 8, 12], contrast use, fluoroscopy and total procedure times [13–15]. Small randomized controlled studies have also demonstrated their potential role in percutaneous coronary intervention training [9, 13, 16]. There have, however, also been studies that have reported contradictory findings, primarily on clinical benefit, adding speculation to their overall advantages [10, 17, 18]. and as a tool for assessing competency.

We, therefore, designed a randomized study examining, as a subject of research, the use of simulation by manipulating a virtual fluoroscopy C-arm with continuous coronary anatomy overlay visualization in addition to a traditional method of learning via a lecture on coronary angiographic views on the achievement of a beginner competency in correct coronary angiographic view interpretation, focusing on early trainees. A beginner competency was defined in this study as an ability to correctly recognize at least 1/3^rd^ of angiographic views correctly. In contrast to prior studies, we decided to focus on a single, important, granular competency with which many early trainees traditionally struggle with and to utilize a function of endovascular simulation which has not been previously well studied – the representation of 3-dimensional virtual anatomy as a teaching tool in addition to standard fluoroscopic 2-dimensional simulated imaging. We hypothesized that the greatest impact of the protocol would be dependent on the level of training and therefore chose to study a convenience sample of medical students, residents and fellows, with a particular emphasis on medical students and residents with either no or little prior exposure to the cardiac catheterization laboratory.

## Methods

### Study population

One hundred and five volunteer trainees, comprising a spectrum of medical students in their third and fourth year of training, internal medicine residents, family medicine residents, emergency medicine residents, and cardiovascular fellows from the University of Arizona met eligibility for the study and were consecutively enrolled from August 2016 to March 2017. Inclusion criteria: 3^rd^ of 4^th^ year medical students, residents from either internal medicine, family medicine or emergency medicine or cardiology fellows at any stage of training at the University of Arizona who provided consent and were willing to participate and complete the training session as well as the pre- and post-test. Exclusion criteria: Trainees outside the defined level of training and who were unable to commit to the training session or testing.

### Ethics

The study protocol was in compliance with the Declaration of Helsinki and was approved by the University of Arizona Human Subject Protection Program Institutional Review Board. All participants were briefed individually at the time of enrollment and they provided written informed consent.

### Study design

At study entry, all participants filled out an online survey to provide data on demographic and baseline self-reported visuo-spatial skills and were assigned a unique identification number at the start of the study. Next, they were administered a self-paced pre-intervention online test (pre-test), (see “Pre-test Coronary Angiographic Training Study”, Supplemental File). This test comprised of 30 de-identified still images and video clips of real angiographic films (that loop automatically every 5 s) in a multiple-choice format. Three questions tested for correct identification of each of the three coronary arteries with three multiple-choice question (MCQ) options. Twenty-seven questions tested for correct identification of the artery and projection with 9 MCQ options covering the six standard angiographic projections for the left coronary artery [left anterior oblique (LAO) caudal, LAO cranial, right anterior oblique (RAO) caudal, RAO cranial], and 3 for the right coronary artery (LAO cranial, AP cranial and RAO).

**Fig. 1:**
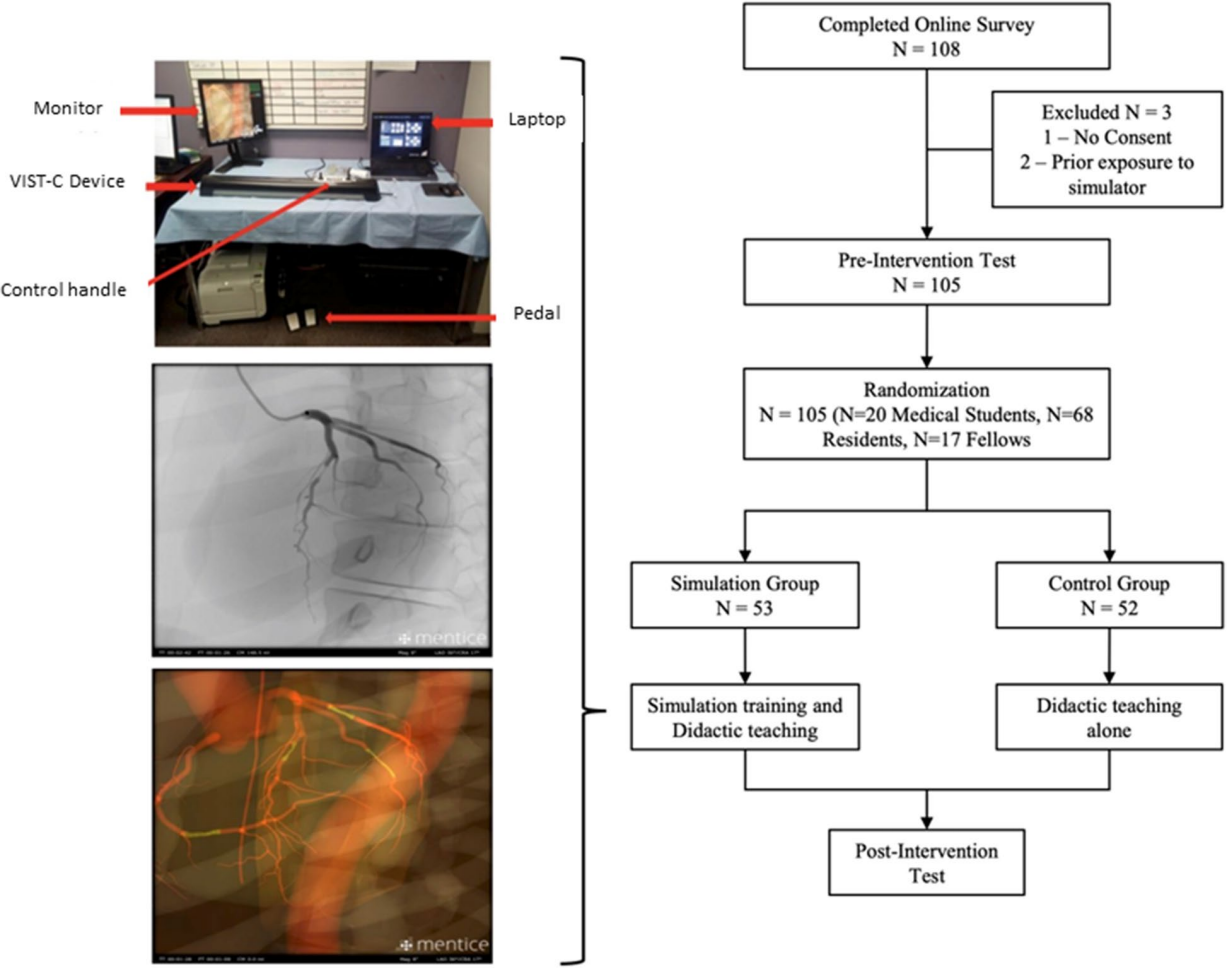
Flow diagram of simulation versus control to teach coronary angiographic interpretation skills. Subjects (medical students, residents, and fellows) were randomly assigned to simulation arm (mentored simulation training using a dedicated simulator with two-dimensional and three-dimensional virtual anatomic views and didactic teaching) versus control (didactic teaching alone with no simulation). Subjects underwent testing before and after training

Study participants were then subjected to a stratified randomization process (Fig. 1) based on their education status (medical student, resident, fellow) and divided into two training arms; a simulation arm which received simulation training in addition to didactic teaching (*N* = 53) and a control arm which received didactic teaching alone (*N* = 52). Blinding was not performed. Convenience sampling was used.

### Simulation interface

The Mentice VIST ® -C (Gothenburg, Sweden) is a portable, high-fidelity endovascular simulator, with good construct and concurrent validity, used in catheter training for coronary and peripheral interventions. This device is connected to a monitor and a laptop which runs the simulation software (VIST-8). The rest of the interface comprises of a dual foot switch for fluoroscopy and cine-angiography, and a syringe for simulated contrast injection (Fig. 1). The simulator includes pre-designed coronary cases with angiographic data, and a single standard case with normal coronary anatomy was selected and used uniformly throughout the study. Additionally, the simulator has buttons and joysticks which enable the operator to virtually move the patient table and the C-arm to switch between angiographic projections akin to a real catheterization laboratory set-up. In addition to simulated standard fluoroscopic imaging, the user is able to switch to 3D visualized virtual anatomy, which overlays the coronary anatomy on the fluoroscopic image.

### Simulation training

Simulation training consisted of a one-on-one mentored training on the simulator for 10–40 min (median: 20 min). The duration of training was decided by the subjects themselves based on their learning pace. At the start of the exercise, they were instructed in person by a trained operator with the aid of a standardized instruction guide to manipulate the table and the C-arm, acquire standard angiographic projections and to recognize dynamic changes in the orientation of the coronary vessels in each of these projections, both in 2D and 3D modes. An instruction manual with conceptual checkpoints and the trained operator were available to the participants throughout the session if needed.

### Didactic teaching

Didactic teaching consisted of printed instruction material and a standardized ten-minute online video tutorial by Morton Kern, MD from his DVD “Cath Lab Essentials with Dr. Morton Kern” on identifying coronary angiographic projections [19]. Subjects could take notes during this session if they desired.

### Evaluation of post-training performance

At the end of the training session, trainees were subjected to the online post-test which comprised of the same 30 projections from the pretest, but randomly shuffled and the test was again self-paced. All pre- and post-test data obtained including test performance scores were archived into an online encrypted folder.

### Statistical analysis

Statistical analysis was performed using STATA version 14.2 (StataCorp., College Station, Texas). All data in this manuscript are represented as mean for parametric continuous variables and as proportions and percentages for categorical variables. Baseline characteristics (Table 1) include a 95% confidence interval for mean age (normal) and for proportions for binomial variables (Wald). ANOVA analysis was used to analyze the effects of training arm and education status (independent variables) and the interaction between these two factors upon the change in test score (delta score). With only two time point measures (pre and post), repeated measures ANOVA was not needed. A two-sided *p* value ≤ 0.05 was considered significant.

**Table 1:** Baseline characteristics

Characteristic	Control group (*N* = 52)	Simulation group (*N* = 53)
Age (years)	30 [29–31]	31 [30–32]
Male gender	60% [46–73]	53% [39–66]
**Visuo-spatial abilities:**		
Play sports	71% [59–83]	55% [41–68]
Play musical instruments	50% [36–64]	40% [26–53]
Play video games	48% [34–62]	34% [21–47]
**Handedness**		
Right	85%	92%
Left	8%	0%
Mixed/ambidextrous	8%	8%
**Preferred Learning Style**		
Visual	46%	60%
Auditory	4%	8%
Tactile	17%	13%
Read/Write	31%	19%
**Stage of Training, N (%):**		
Med Student (YR 3&4)	21%	17%
PGY-1 Resident	23%	23%
PGY-2 Resident	23%	26%
PGY-3 Resident	17%	17%
PGY-4 CV Fellow	6%	8%
PGY-5 CV Fellow	6%	6%
PGY-6 CV Fellow	4%	4%
**How confident are you with your knowledge on normal coronary anatomy?**		
1 (Not confident at all)	12%	11%
2 (slightly confident)	33%	30%
3 (somewhat confident)	40%	43%
4 (fairly confident)	15%	13%
5 (completely confident)	0%	2%
**How would you grade your knowledge on coronary angiography?**		
Novice	29%	32%
Beginner	62%	55%
Intermediate	8%	11%
Advanced	2%	2%
**Can you interpret coronary angiographic images accurately?**		
No	73%	59%
Somewhat	11 (21%)	21 (40%)
Yes	3 (6%)	1 (2%)

## Results

### Study population characteristics

A total of 105 subjects were enrolled in a convenience sample, comprising *N* = 20 medical students, *N* = 68 residents and *N* = 17 cardiology fellows. Participants were predominantly male (59%) with a mean age of 30 ± 4 years. Among the 68 residents, 55 were from Internal Medicine, 6 from Family medicine and 7 from Emergency medicine. A majority, 84%, of the participants self-reported playing a musical instrument, competitive sports, or video game routinely over the past year. In terms of handedness, the majority were right-handed (89%) with a small number of them reporting mixed handedness or being ambidextrous (8%). Prior to taking the study, 89% of the participants graded themselves as a novice or a beginner in coronary angiographic training of which 96% of them stated that they were not confident in accurately identifying coronary anatomy on an angiographic image. There were no significant differences in these baseline characteristics between the simulation and control arms (Table 1). After completion of the training experience, 50% rated the lecture as good or excellent. Among those randomized to simulation, 85% graded their satisfaction with the simulation experience as good or excellent and 98% agreed or strongly agreed that simulation training is essential in cardiovascular fellowship training. There were no adverse events.

### Test performance scores

**Fig. 2:**
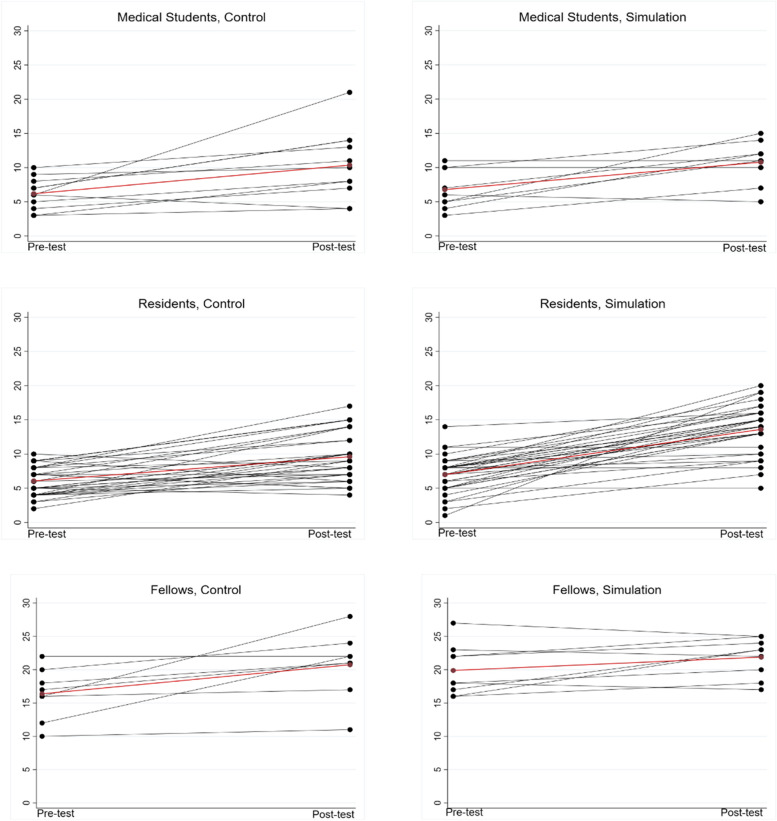
Pre- and Post-test scores by education status and training group. Line plots of pre- and post-test scores for medical students (top row), residents (middle row) and fellows (bottom row), by control group (left panel) and simulation group (right panel). Mean values of pre- and post-test scores shown in red

Out of a maximum score of 30, the pre-test score was 6.5 ± 2.5 for medical students, 6.5 ± 2.5 for residents, and 18.2 ± 4.1 for fellows (*p* < 0.0001 for effect of education status). The change in score (delta score) from pre- to post-test is given in Table 2. Subjects improved in their post-test scores with a mean delta score of 4.6 ± 4.0, with subjects that underwent simulation training having a greater delta score (5.4 ± 4.2 vs 3.8 ± 3.7, *p* = 0.04). The interaction of factors of training arm (simulation versus control) and education group (medical student, resident, fellow) were further evaluated in a two-way ANOVA. Education status alone was not significant (*p* = 0.13). However, there was a significant interaction effect of training arm and education status (*p* = 0.02), such that residents derived the greatest benefit from simulation training compared to control (6.6 ± 4.0 versus 3.5 ± 3.4, Table 2). Pre- and post-test scores by training arm and education status are shown in Fig. 2.

**Table 2:** Difference in test score after control or simulation training according to education group

	All Training Arms	Control (*N* = 52)	Simulation (*N* = 53)^a^
All education groups	4.6 ± 4.0	3.8 ± 3.7	5.4 ± 4.2
Medical Student (*N* = 20)	4.1 ± 4.1	4.2 ± 4.5	4.0 ± 3.8
Resident (*N* = 68)^b^	5.1 ± 4.0	3.5 ± 3.4	6.6 ± 4.0
Fellow (*N* = 17)	3.1 ± 3.8	4.4 ± 4.4	2.0 ± 3.1

^a^ *p* = 0.04 for training arm

^b^ *p* = 0.02 for interaction effect of training arm and education group, with residents showing the greatest positive impact of simulation

The delta scores by angiographic view are shown in box plots in the Supplemental Fig. 1. In this figure there is a visual impression that simulation best improved the identification of the RAO projections of the right and left coronary arteries and of the LAO cranial projection of the right coronary artery.

## Discussion

The results of this study demonstrate that one brief (median 20-min) training session using a high-fidelity three-dimensional simulation module, when used to supplement traditional lecture-based learning can significantly accelerate early trainees’ attainment of the competency to correctly identify coronary angiographic projections. Although the effect was modest, the amount of time investment involved relative to the gain in knowledge should be noted as it sometimes takes weeks to months to achieve the same level via traditional learning. Manipulating the C-arm in simulations allows the trainee to explore virtual three-dimensional coronary anatomies actively in real-time, thereby facilitating internal mental anatomical model construction, developing hand–eye coordination skills, and improving confidence in troubleshooting technical challenges in a safe learning environment. The ability to continuously track the coronary arteries in these simulation training sessions is a distinct advantage in visual-spatial learning compared to traditional interrupted 2-dimensional representation of coronary anatomy between shots in real world angiography.

Prior studies have consistently shown the greatest impact of simulation in novice trainees, consistent with findings reported from other simulation-based studies [9, 16, 20, 21]. Correspondingly, the greatest improvement in our study was noted in early trainees, specifically residents, which are best representative of a new cardiology fellow with no prior cath lab experience. These findings further support the need for more studies to justify the adoption of a simulation curriculum early on in undergraduate and graduate medical education programs [22].

The discrepancy between cardiology fellows and novice trainees is likely explained by the fellows’ previous attainment of the tested competency (basic anatomical identification on coronary angiograms) during their clinical training and experience. The study was conducted later in the academic year, and even our first-year cardiology fellows had already been exposed to coronary angiographic interpretation. Therefore, our described simulation training methodology should be integrated into a curriculum as part of introductory training. More broadly, our work demonstrates the importance of targeting a training protocol to the appropriate trainee. We speculate that cardiology fellows would best benefit from more advanced training protocols, such as teaching how to anticipate C-arm positioning to best visualize coronary anatomy.

To our knowledge, this is the first randomized controlled study to investigate the additive role of high-fidelity simulation training to traditional methods in teaching basic coronary angiography view interpretation to junior physicians. A recent study from France by Fischer et al [23] randomized 118 medical school students into simulation and traditional power-point based teaching. They reported that the simulation group did better in identifying coronary anatomy and coronary angiographic projections after a single simulation session. Although our main findings were similar, our study design is different. Firstly, we recruited a spectrum of trainees at various levels of clinical training, beyond medical students, to examine if more experienced trainees would benefit. Next, in order to allow for different learning speeds and preferences, subjects in the simulation arm were provided one-on-one instruction at the start of the exercise and then allowed independent unobserved practice time with no restriction on the amount of time spent on the simulator. Finally, since our subjects had varying amounts of exposure to clinical cardiology and familiarity with coronary angiography, we decided to focus on improvement in test performance from baseline, pre-intervention to post-intervention (delta scores) as our major primary outcome rather than an isolated post-intervention score by itself as reported by Fischer et al [23].

### Limitations

There are limitations to our study that are inherent with our sample size and study design. Our results for fellows are likely affected by their small sample size and varied amount of exposure to CA prior to the study. However, a study specific to cardiology fellows would require a multi-year and multi-center study, which would be limited by the general availability of coronary simulators. We did not perform quantitative assessment of baseline visuospatial skills of our study participants and so their influence if any on the study outcome is unknown. We were also unable to explore the effect of a single structured simulation session on long-term retention. Also, the additive effect of periodic booster training sessions on knowledge acquisition and retention was not studied. It would have been interesting to see if subjects in the control arm would have benefitted from crossing over to simulation training at the end of the study by administering a repeat assessment. This study was not blinded, but as the outcomes measurement was the performance on a multiple choice question test, the lack of blinding is unlikely to have caused bias. There was no sample size calculation according to a predetermined improvement in performance.

## Conclusion

In conclusion, this study highlights the growing role of simulation based medical education in the field of cardiology beyond just acquiring procedural skill in the cath lab. It suggests that even a single, brief targeted training session can rapidly improve early trainees’ attainment of coronary angiographic projection competency. This study strengthens the case for the development of a framework for learning and competency assessment using simulation. Further large studies are needed to justify the cost of implementing simulation-based programs as part of cardiovascular fellowship training and should focus on tailored simulation training methodology for achieving specific competencies. Despite the cost barriers of integrating simulation in training, widespread adoption will hopefully result in a decrease in cost via the economies of scale.

## Supplementary Information

**Additional file 1.**

## Data Availability

The datasets used and/or analysed during the current study available from the corresponding author on reasonable request.
