# Supplementary material for: A randomized controlled trial of simulation training in teaching coronary angiographic views
Source: BMC Med Educ. 2022 Aug 26;22:644. doi: 10.1186/s12909-022-03705-z (PMC9414435; doi:10.1186/s12909-022-03705-z)

**SUPPLEMENTARY FILE**

1. “Pre-test Coronary Angiographic Training Study”

2. Supplementary Figure 1. Box plots demonstrating delta scores by angiographic views.


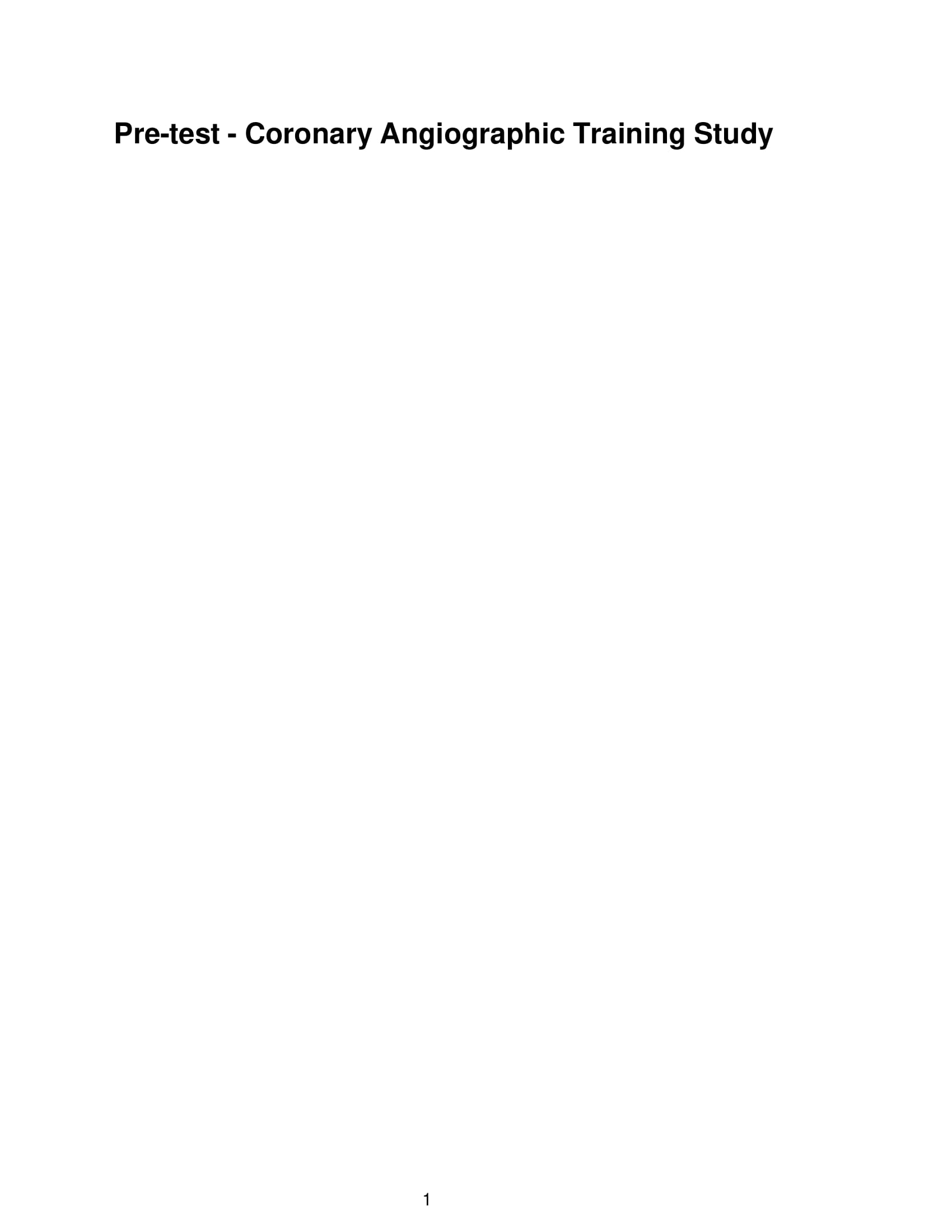


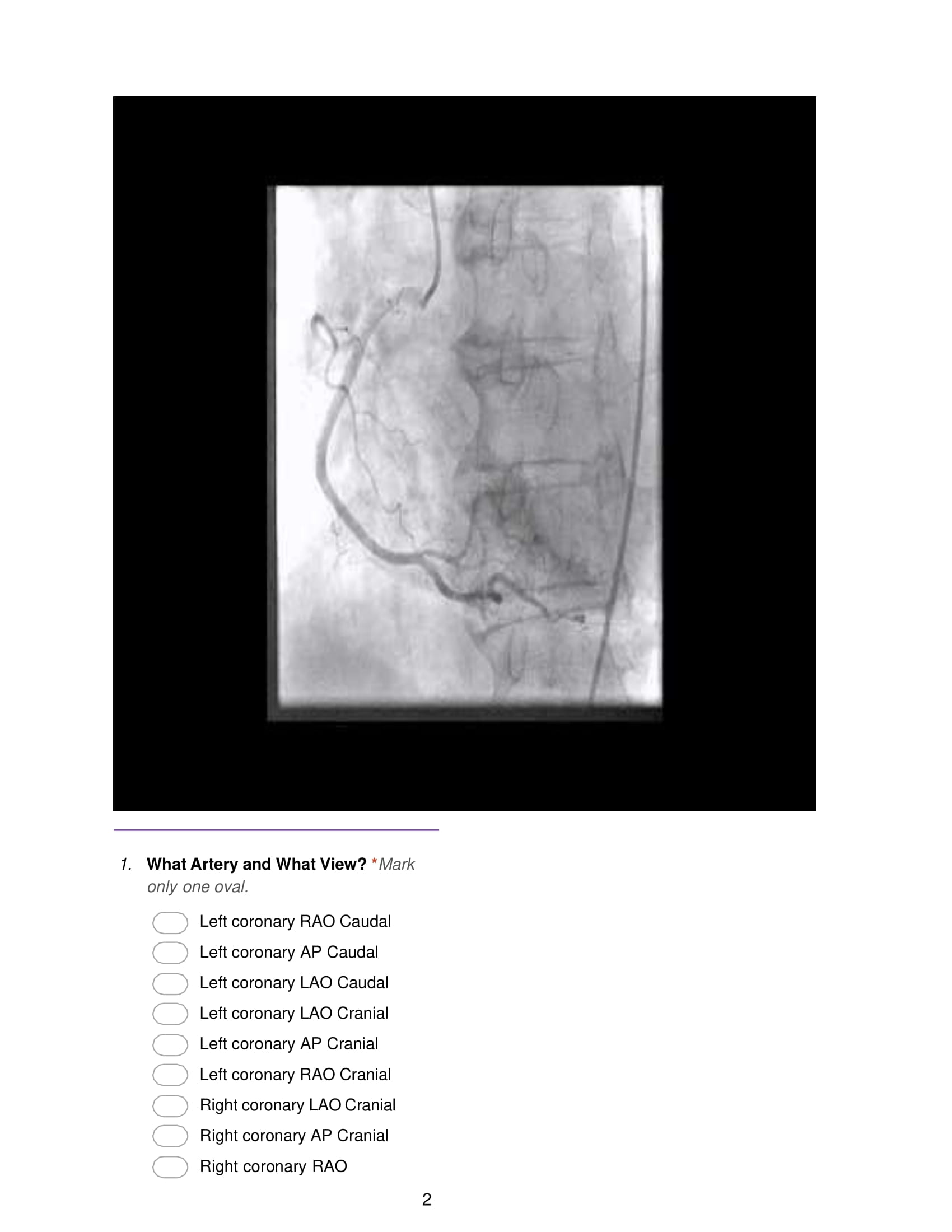

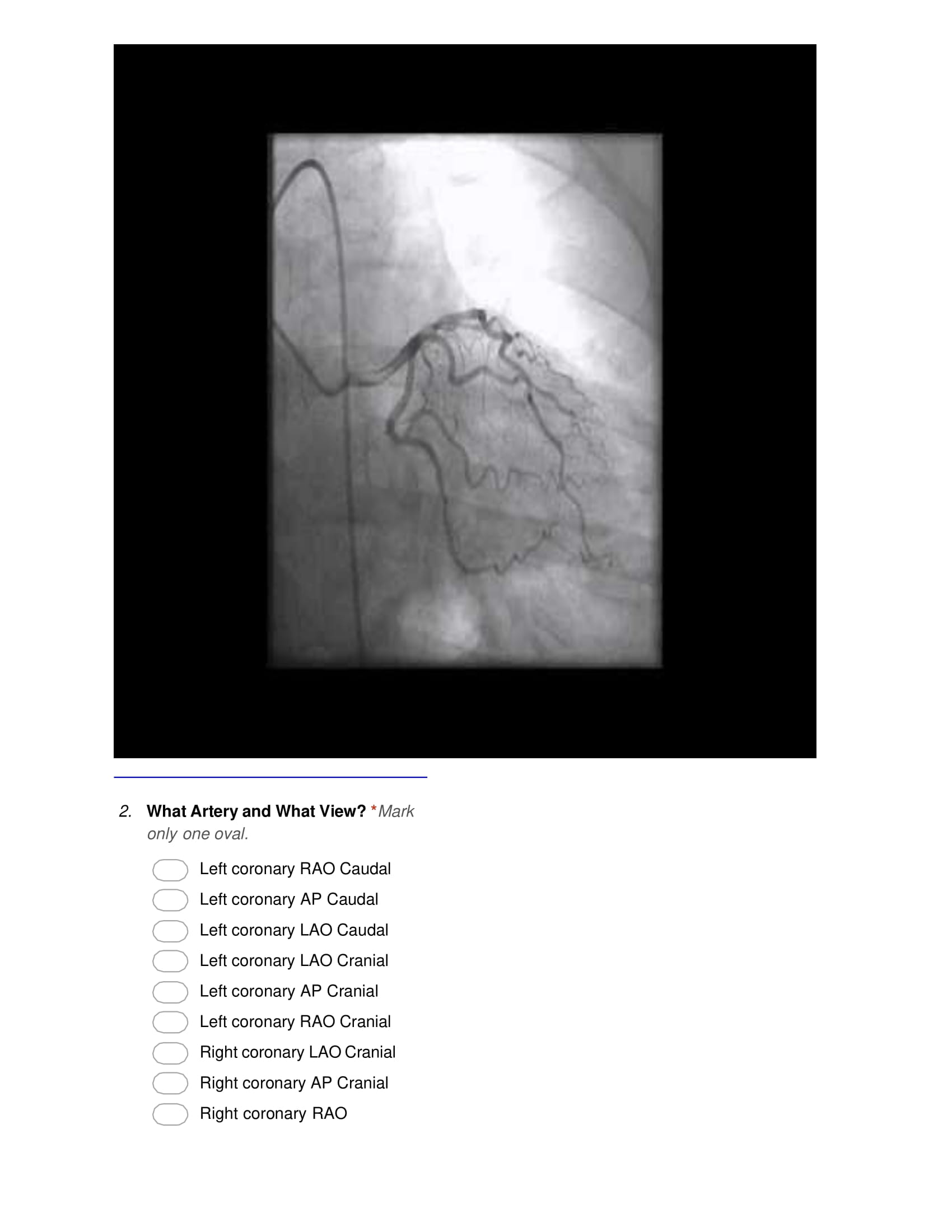

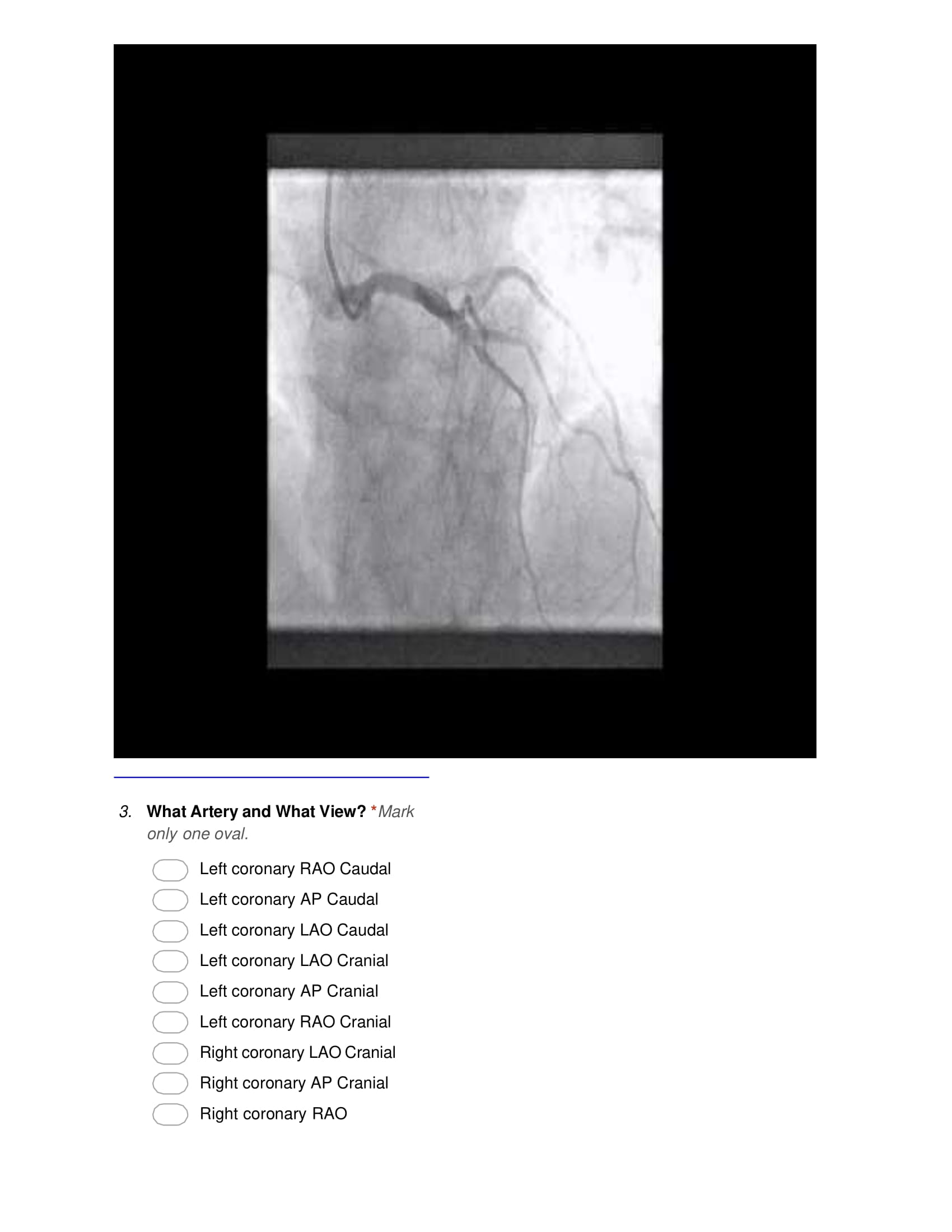

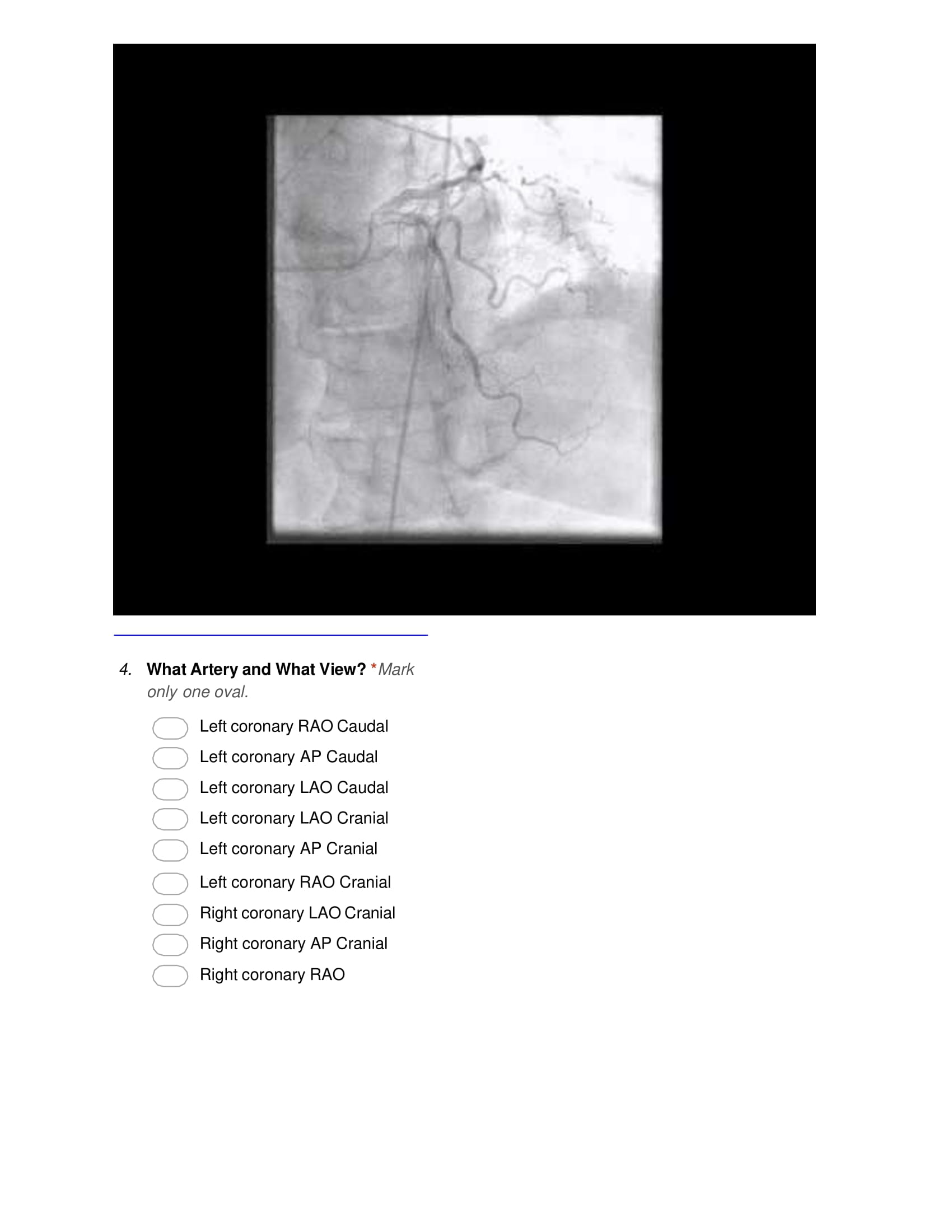

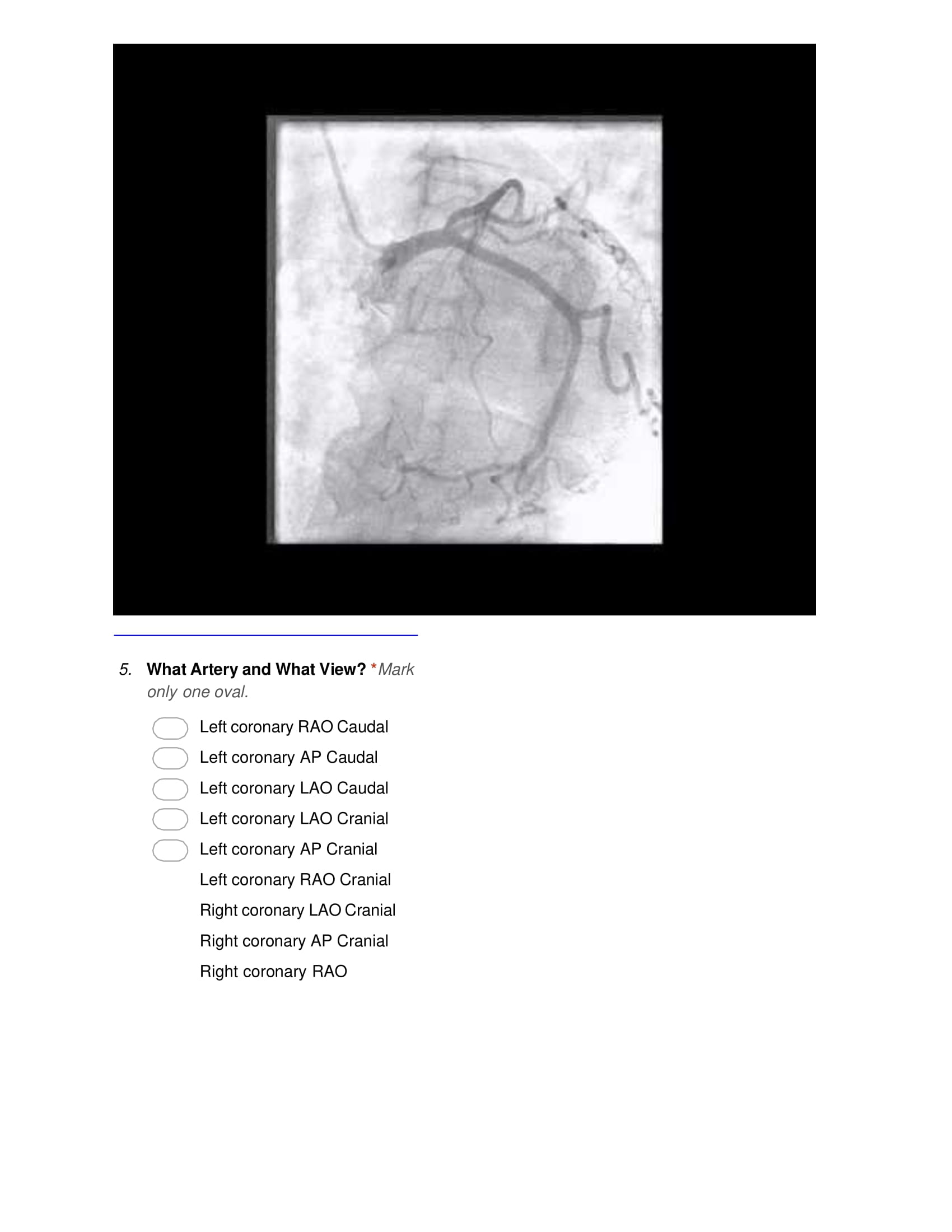

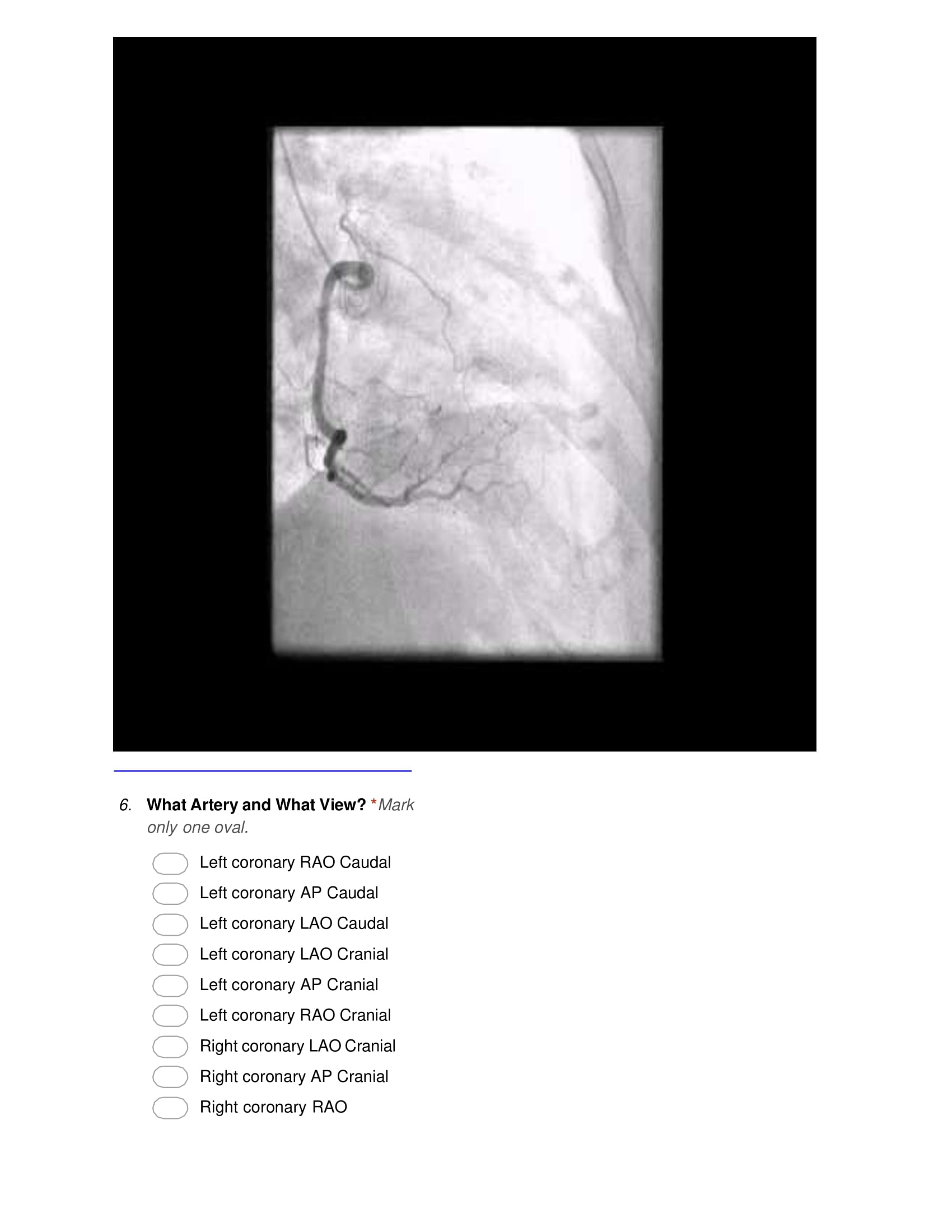

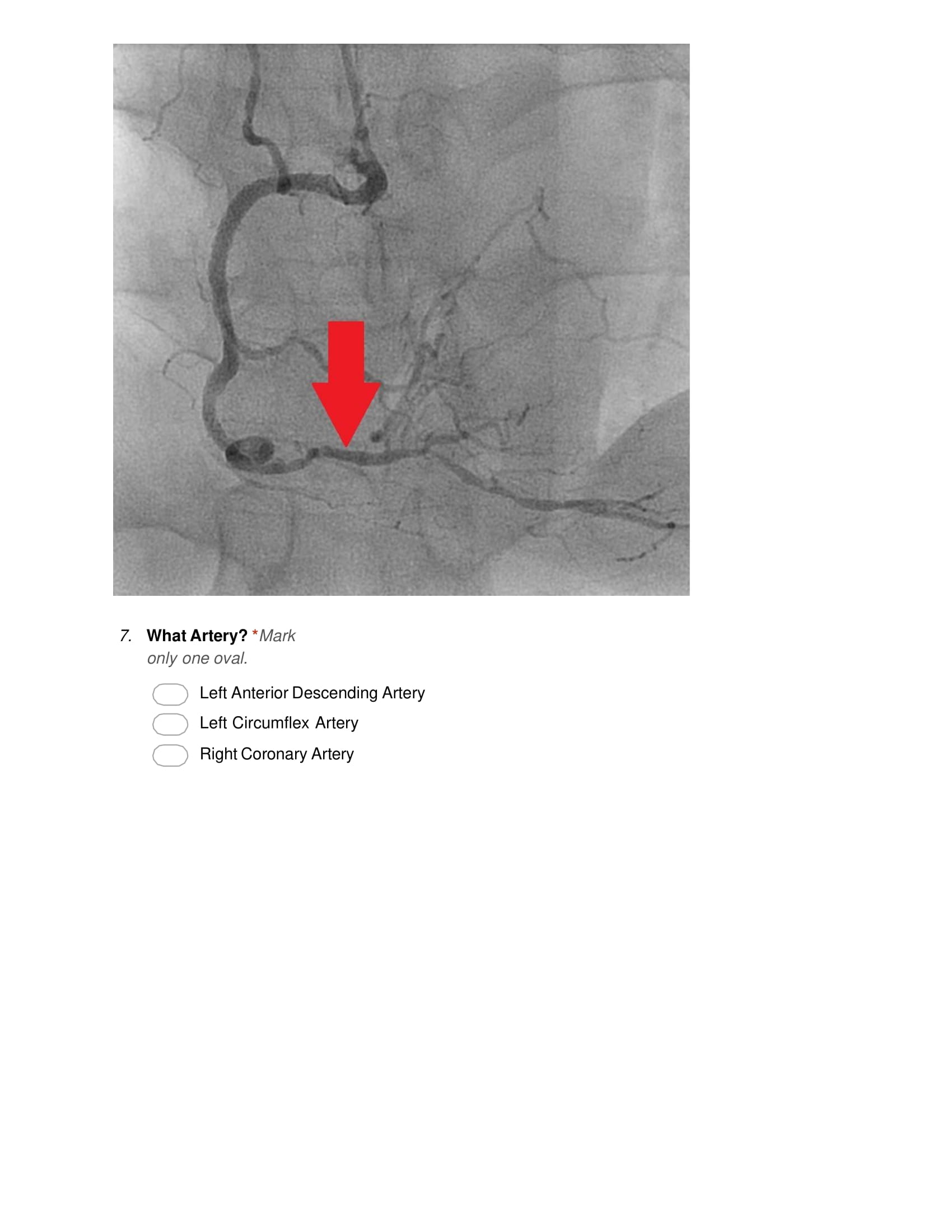

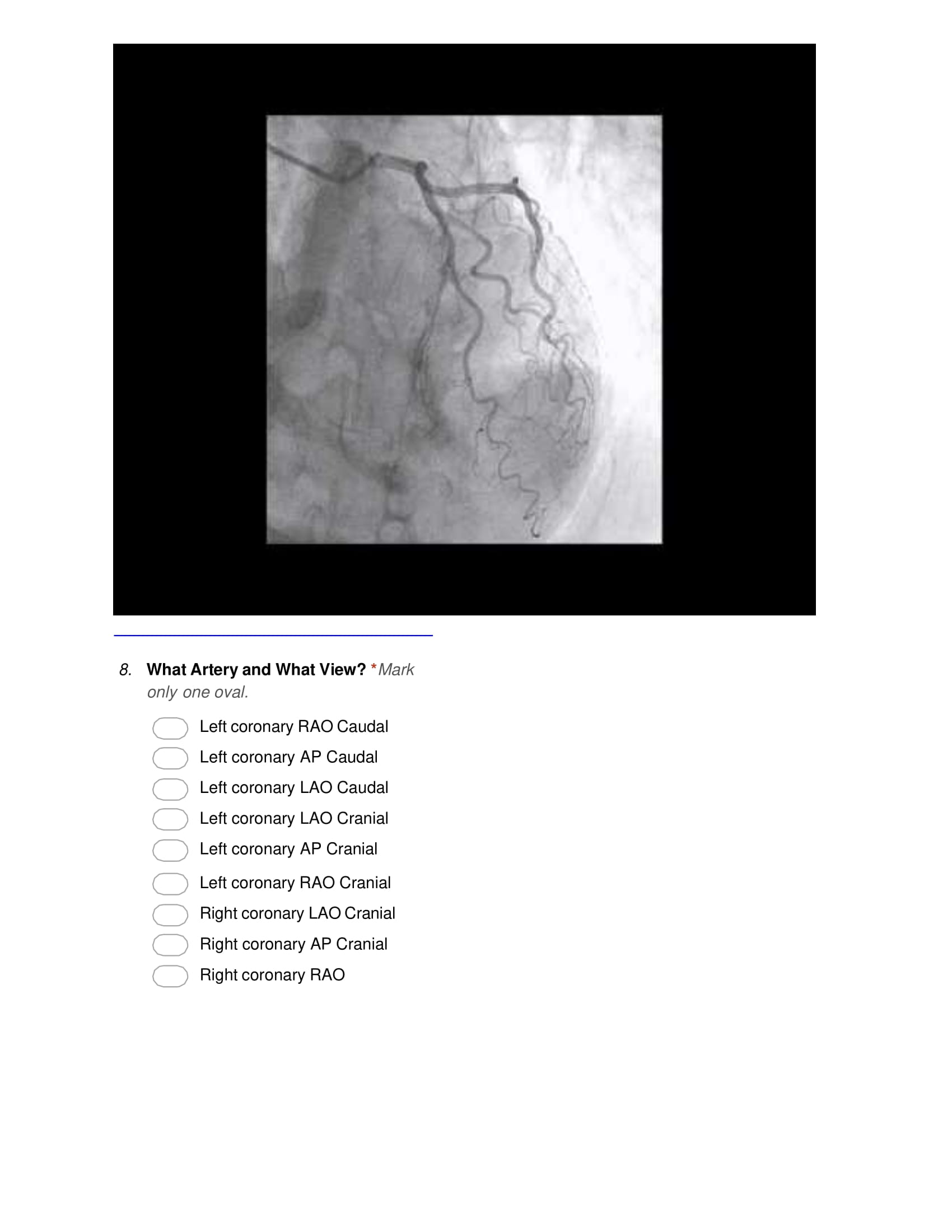

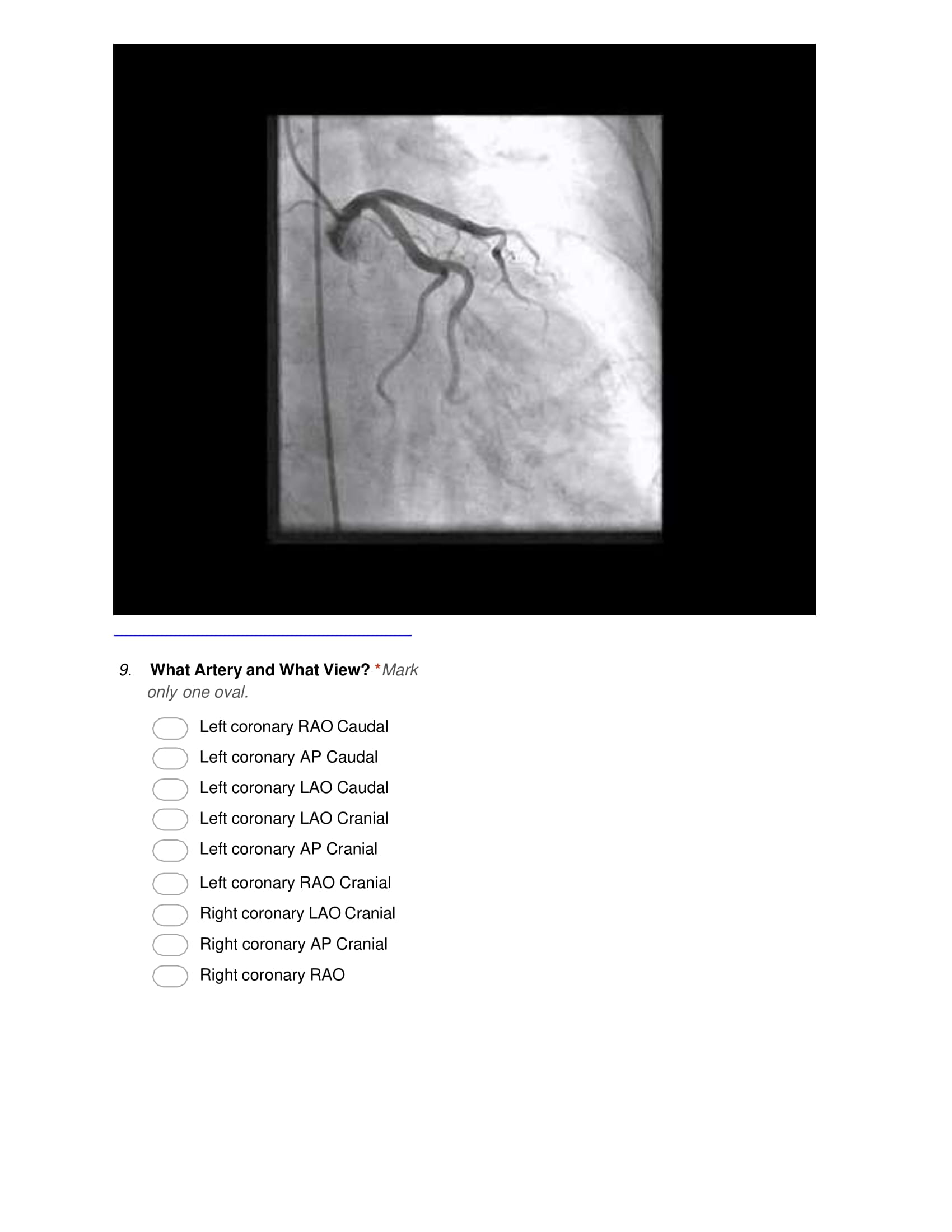

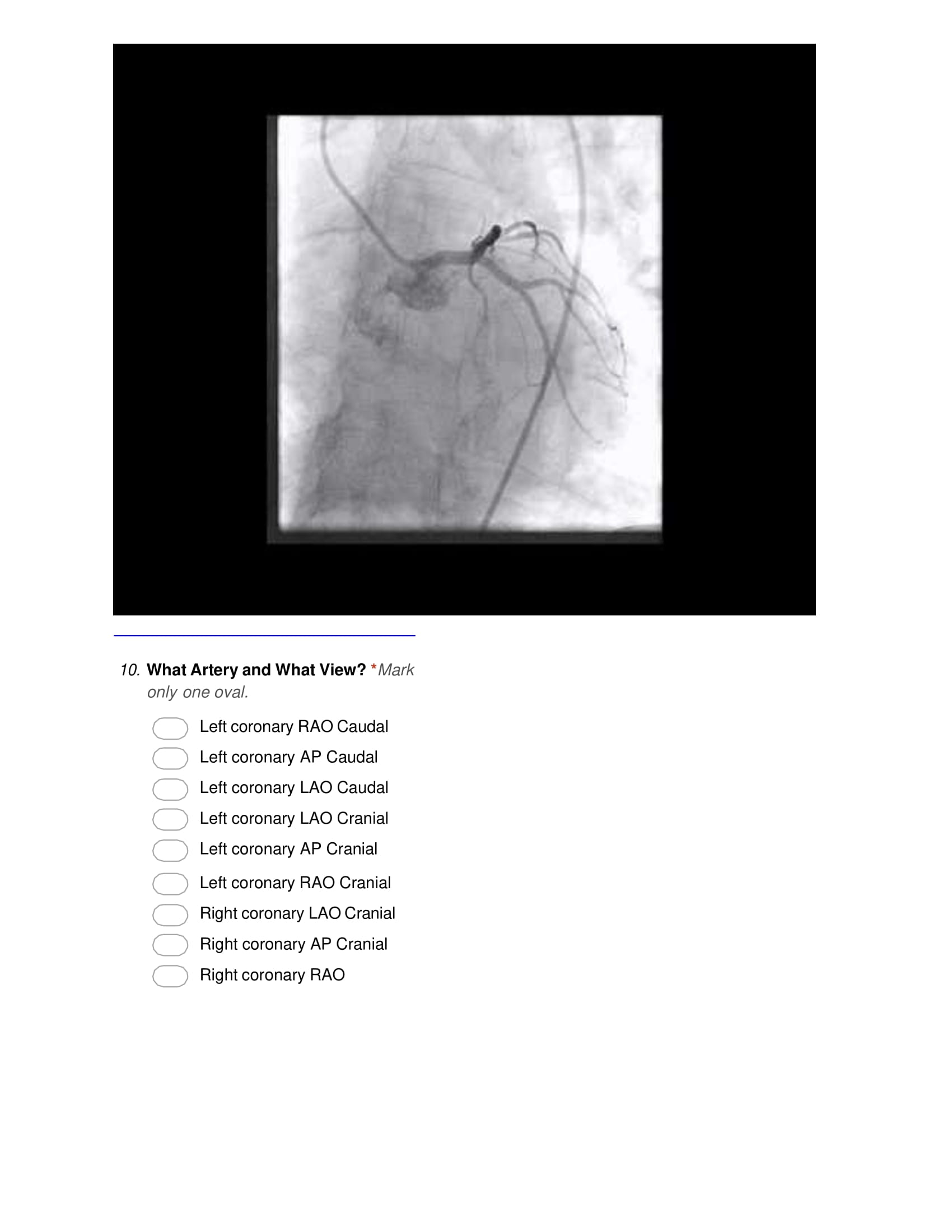

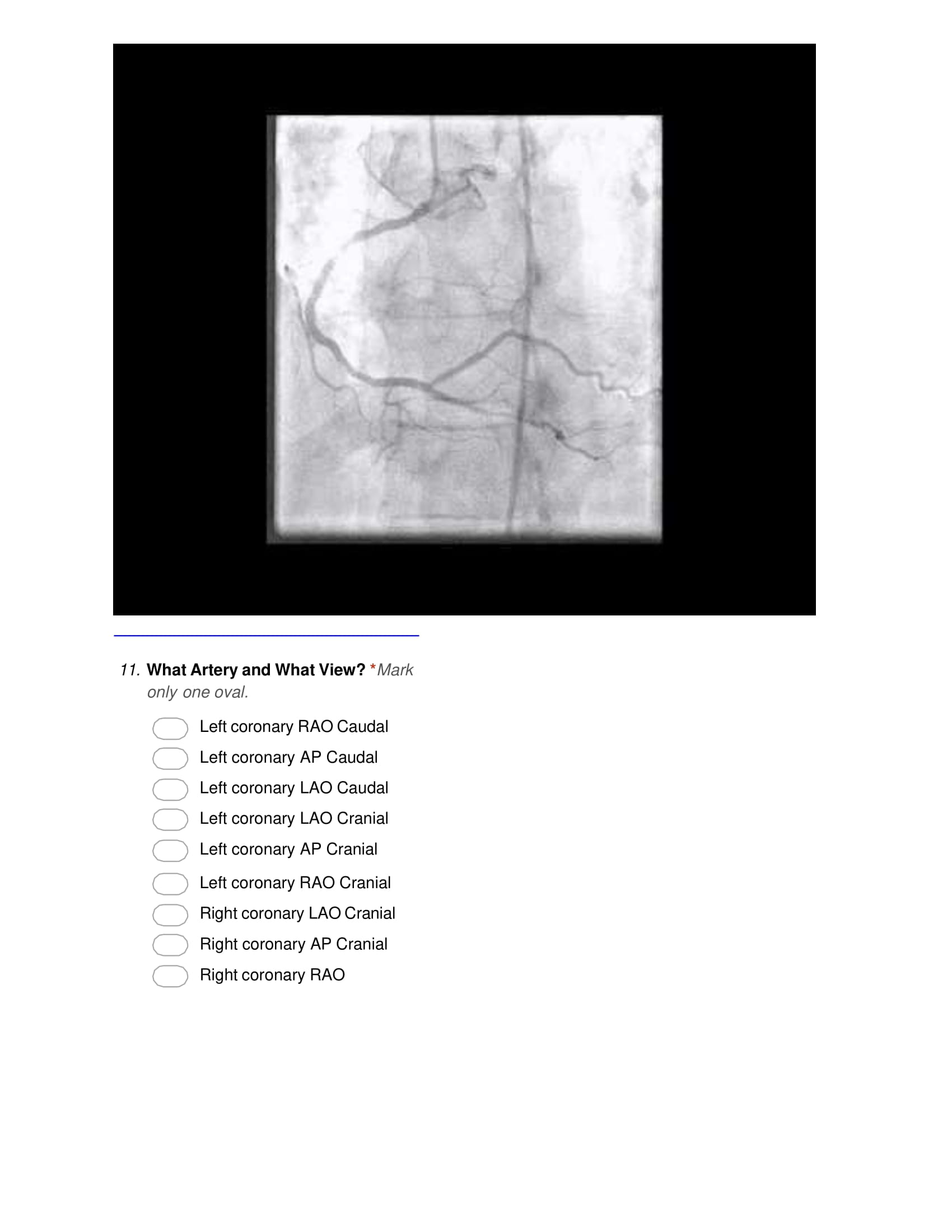

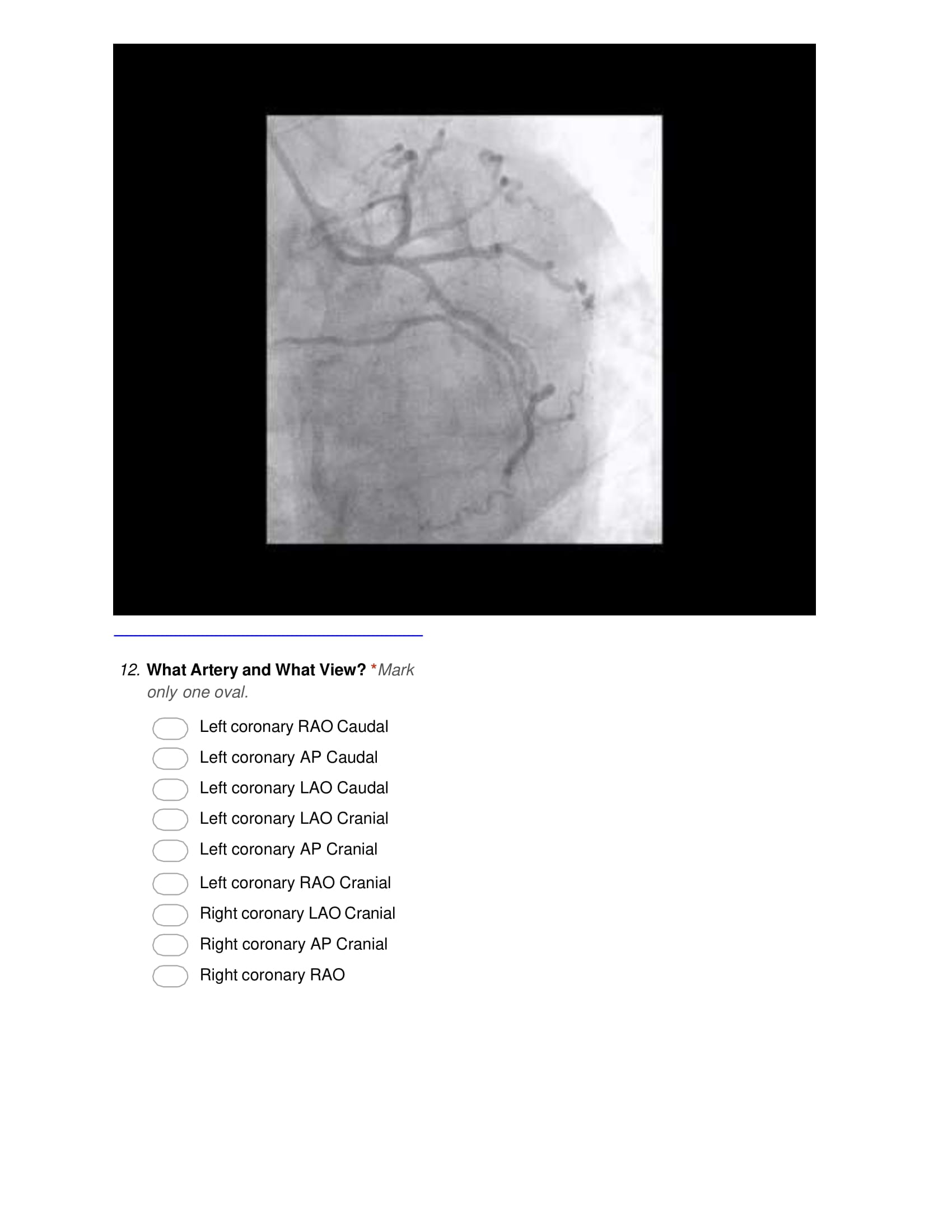

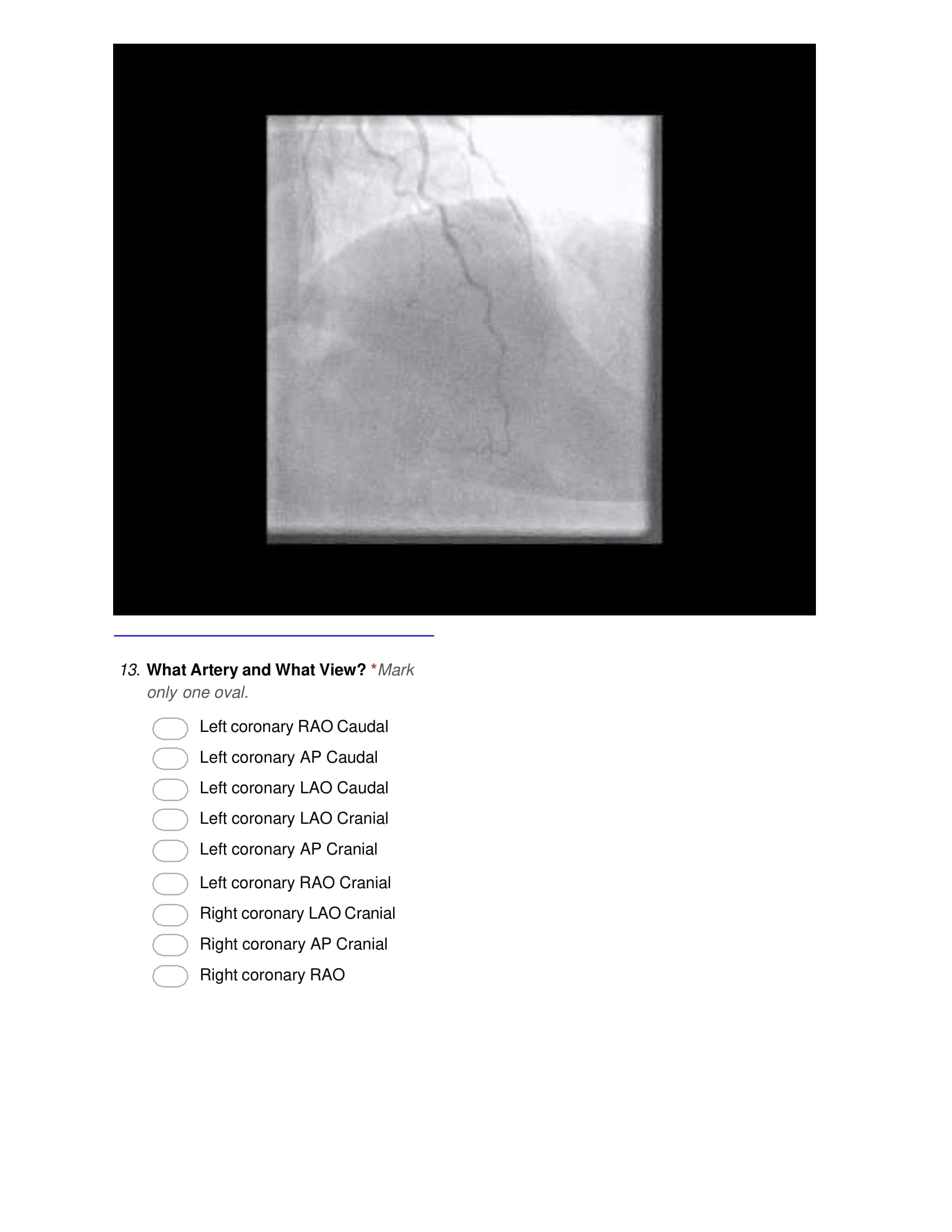

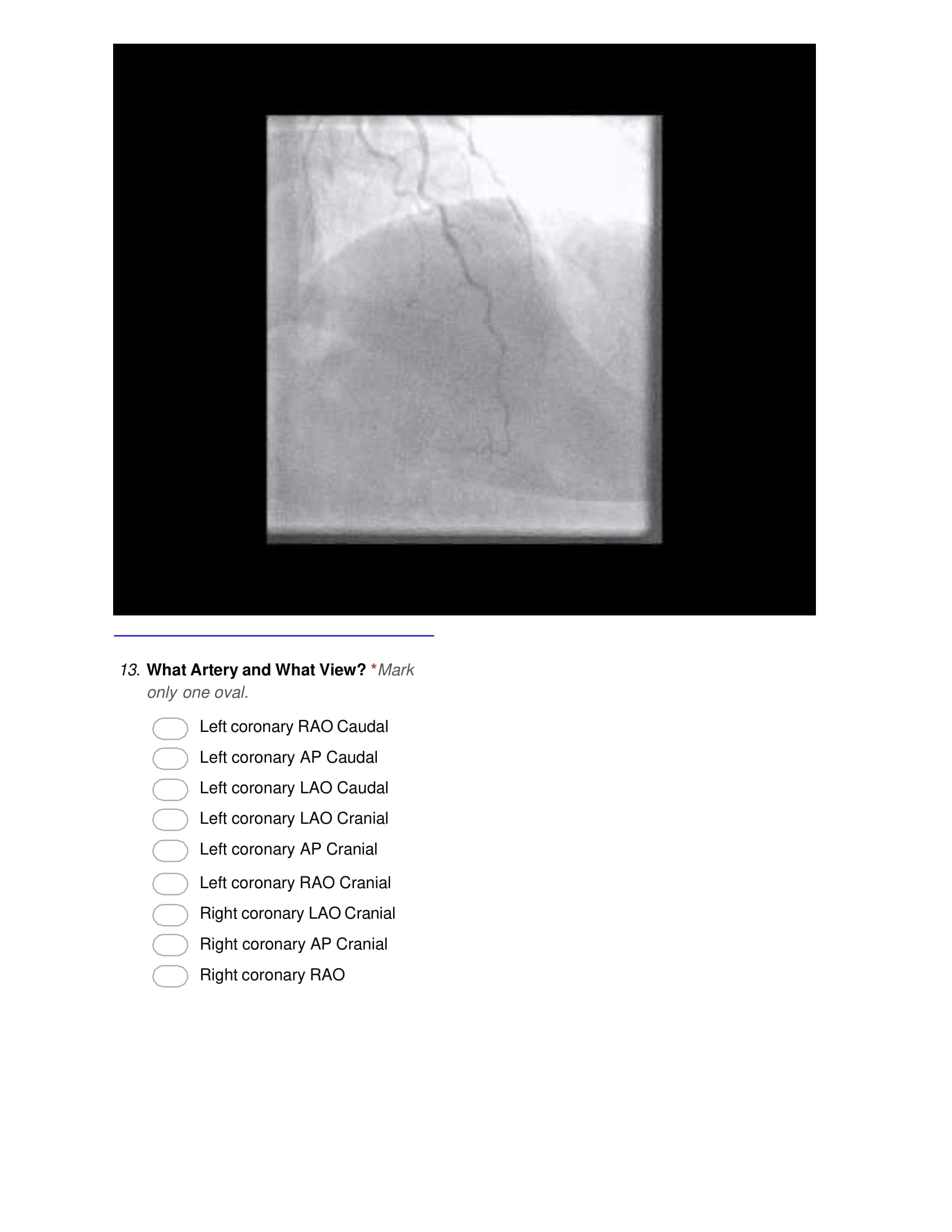

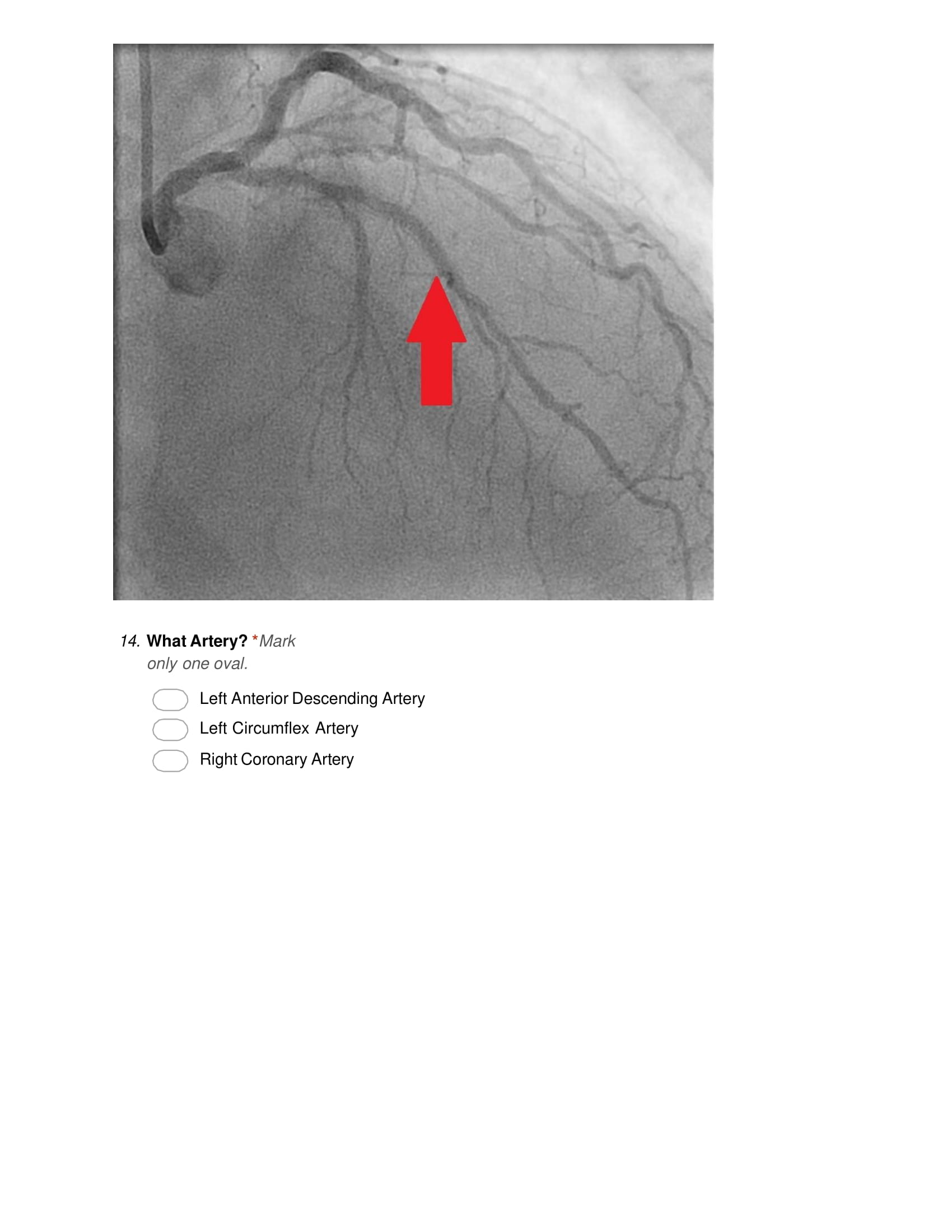

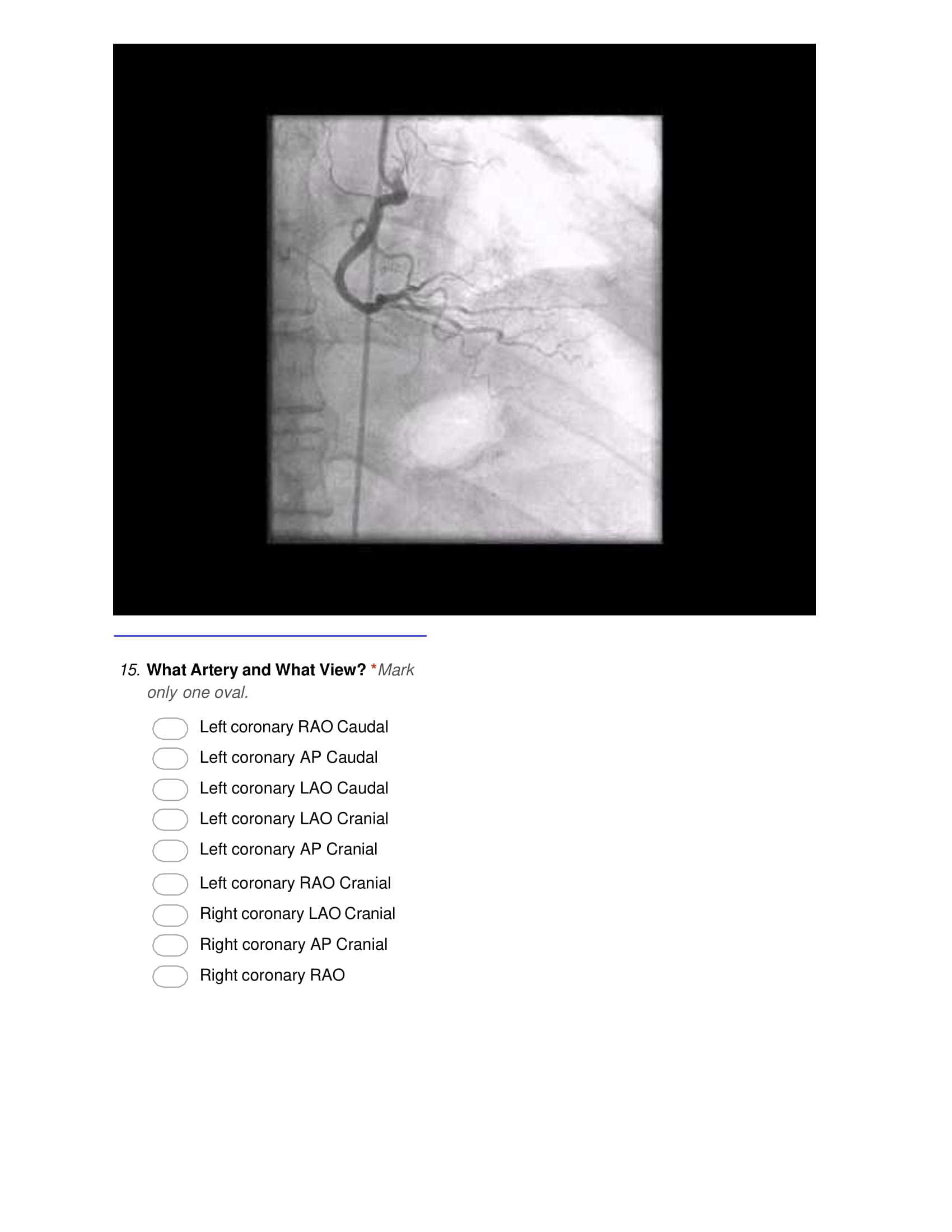

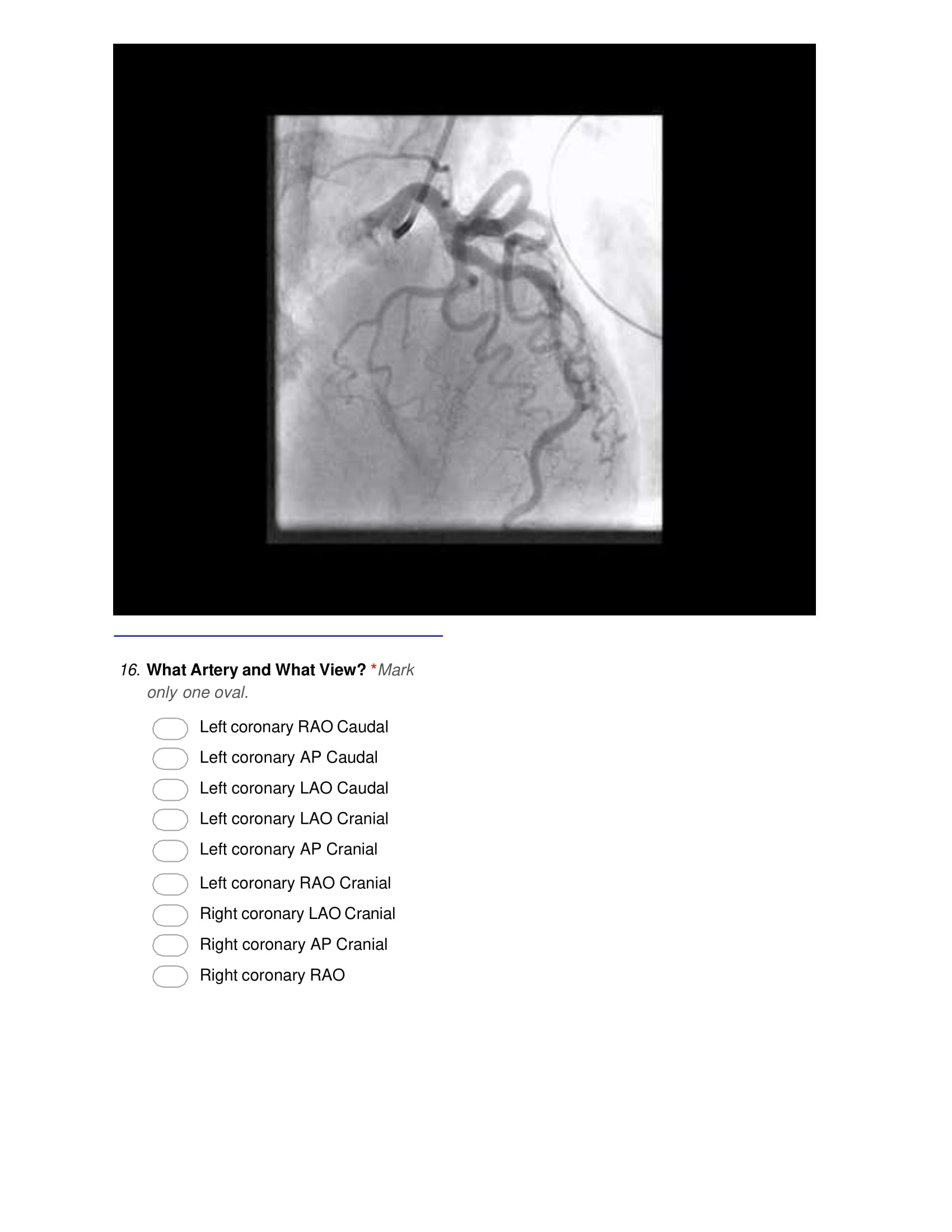

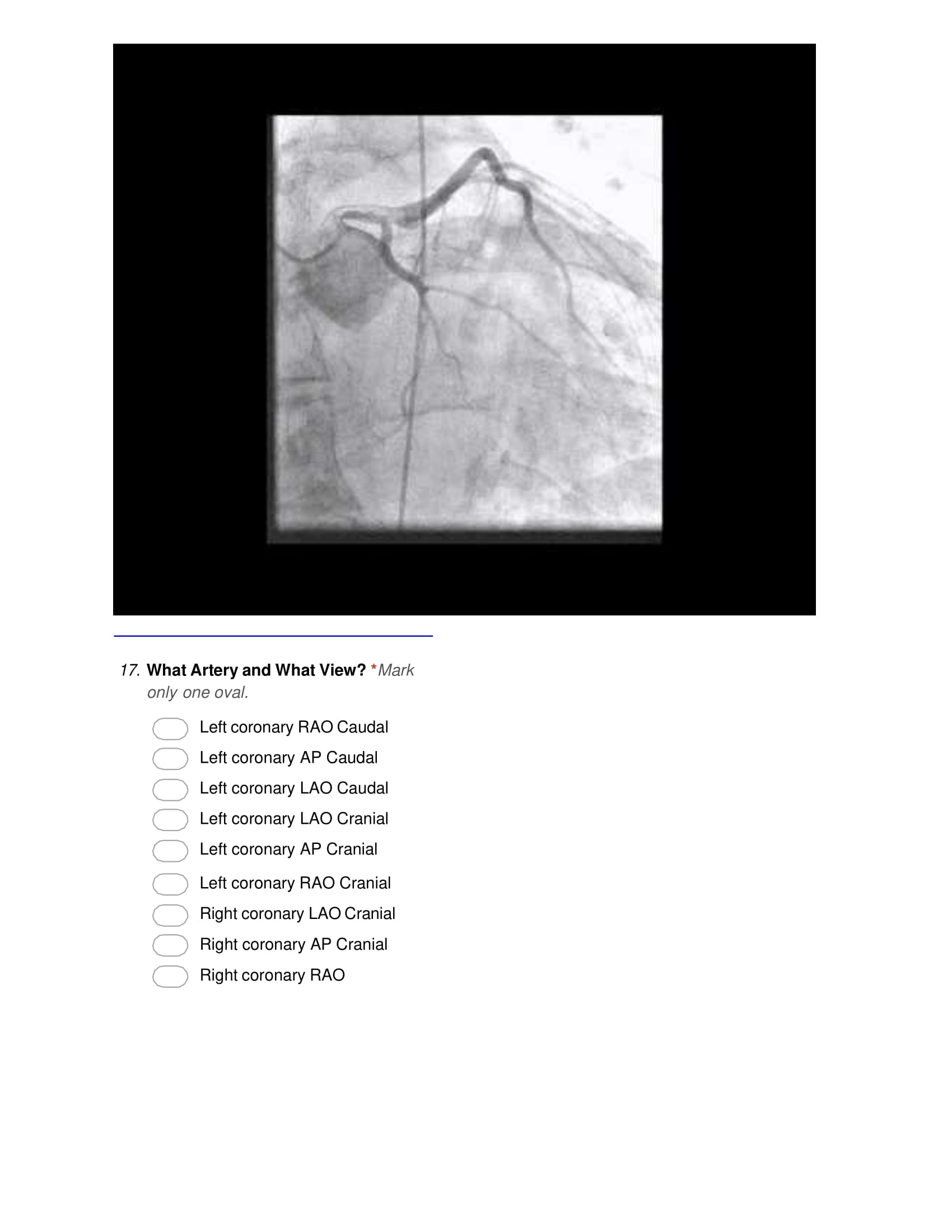

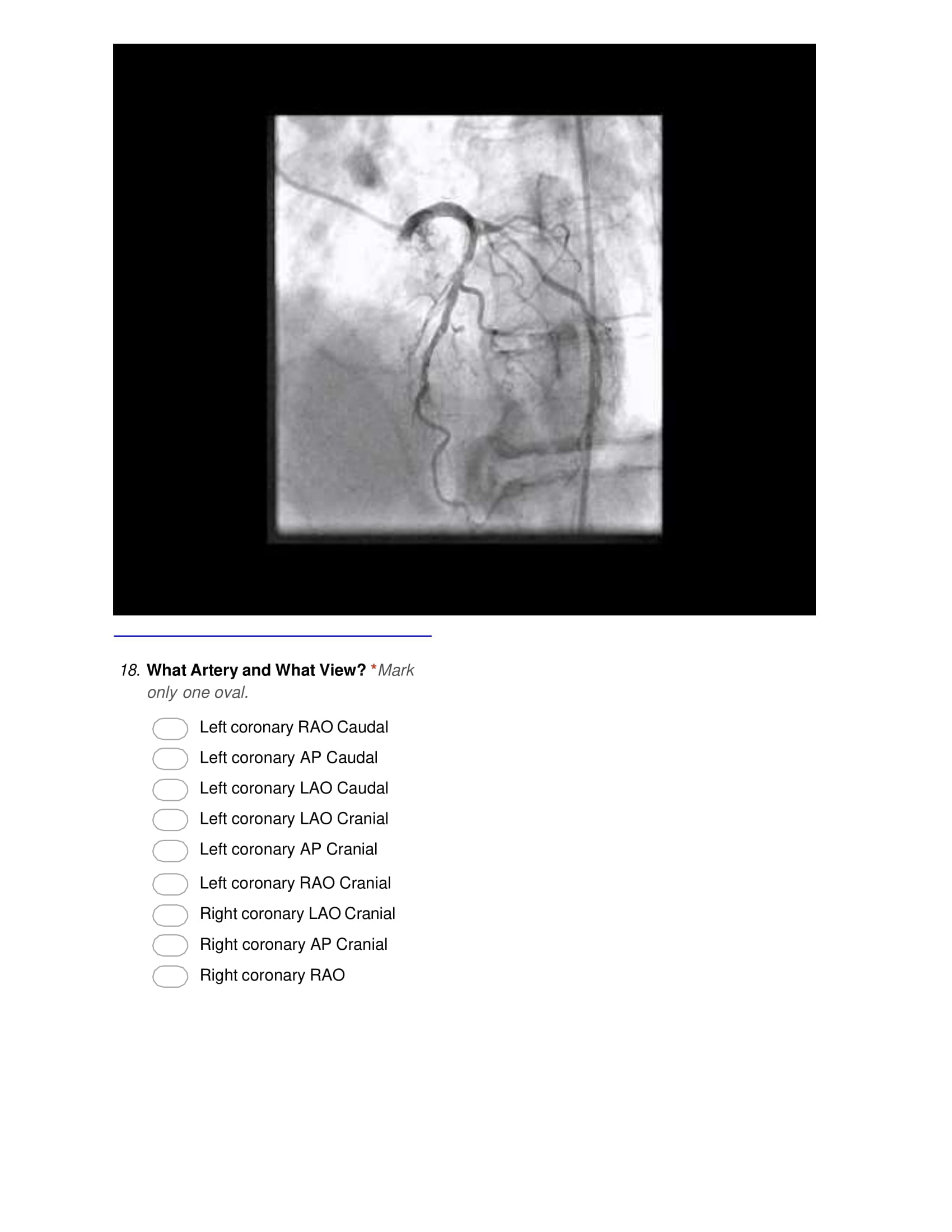

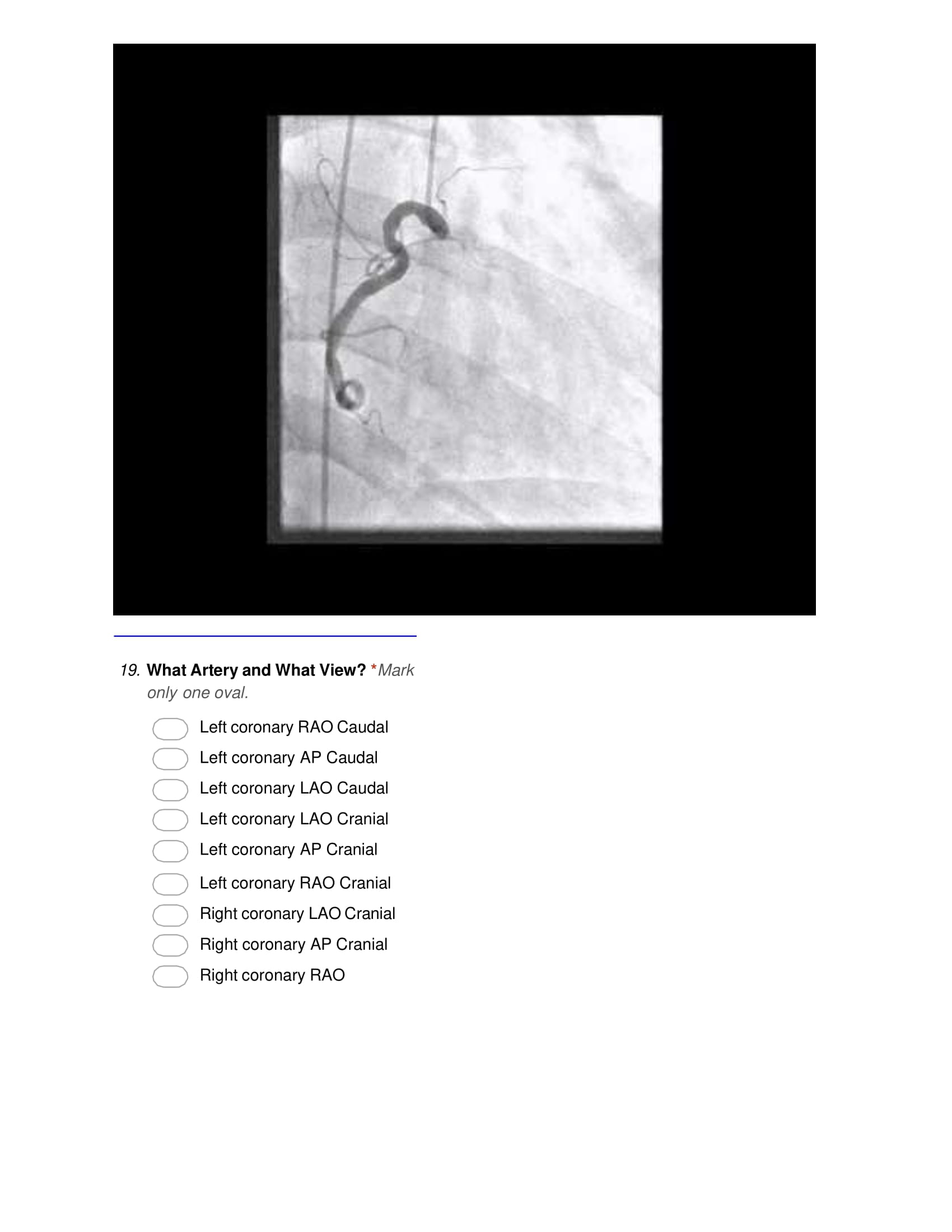

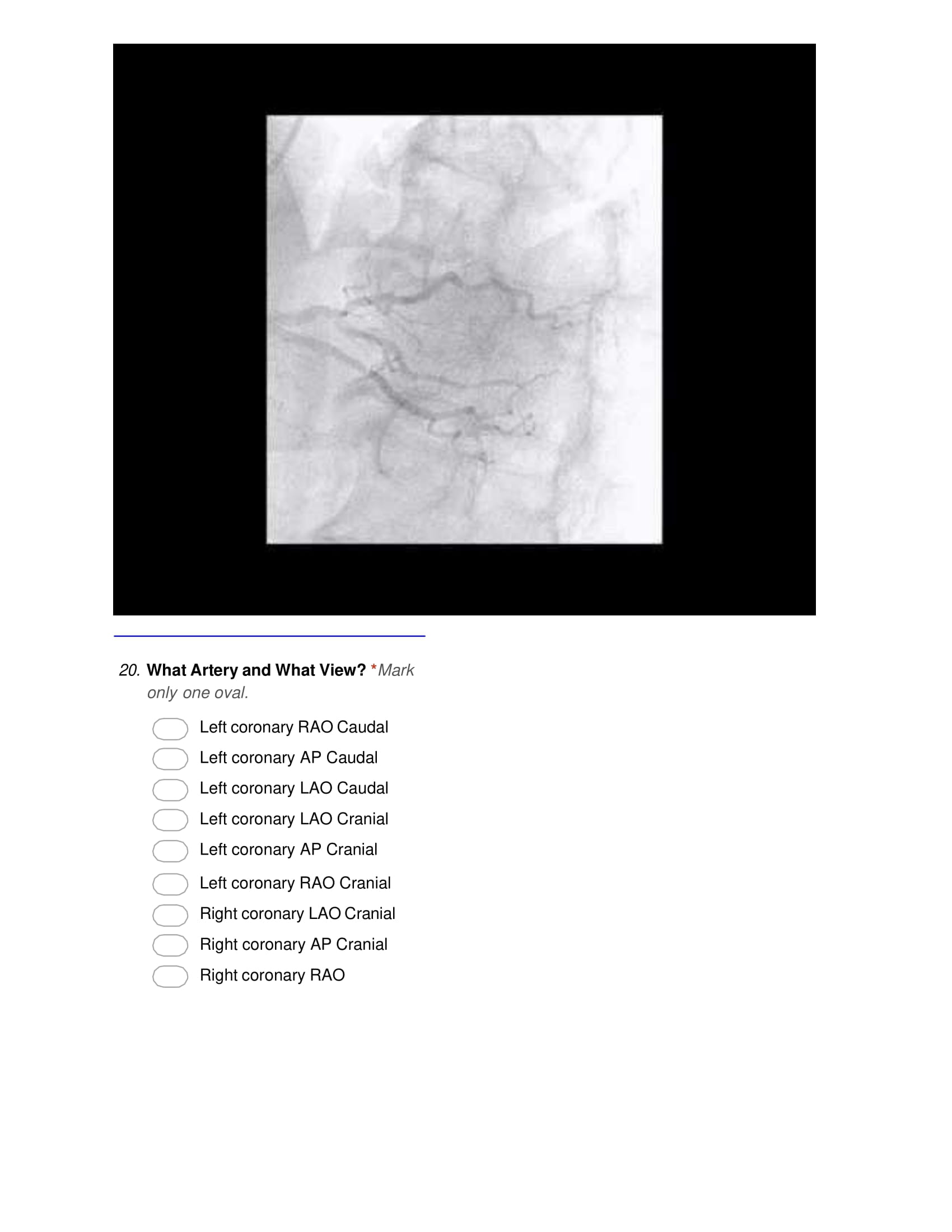

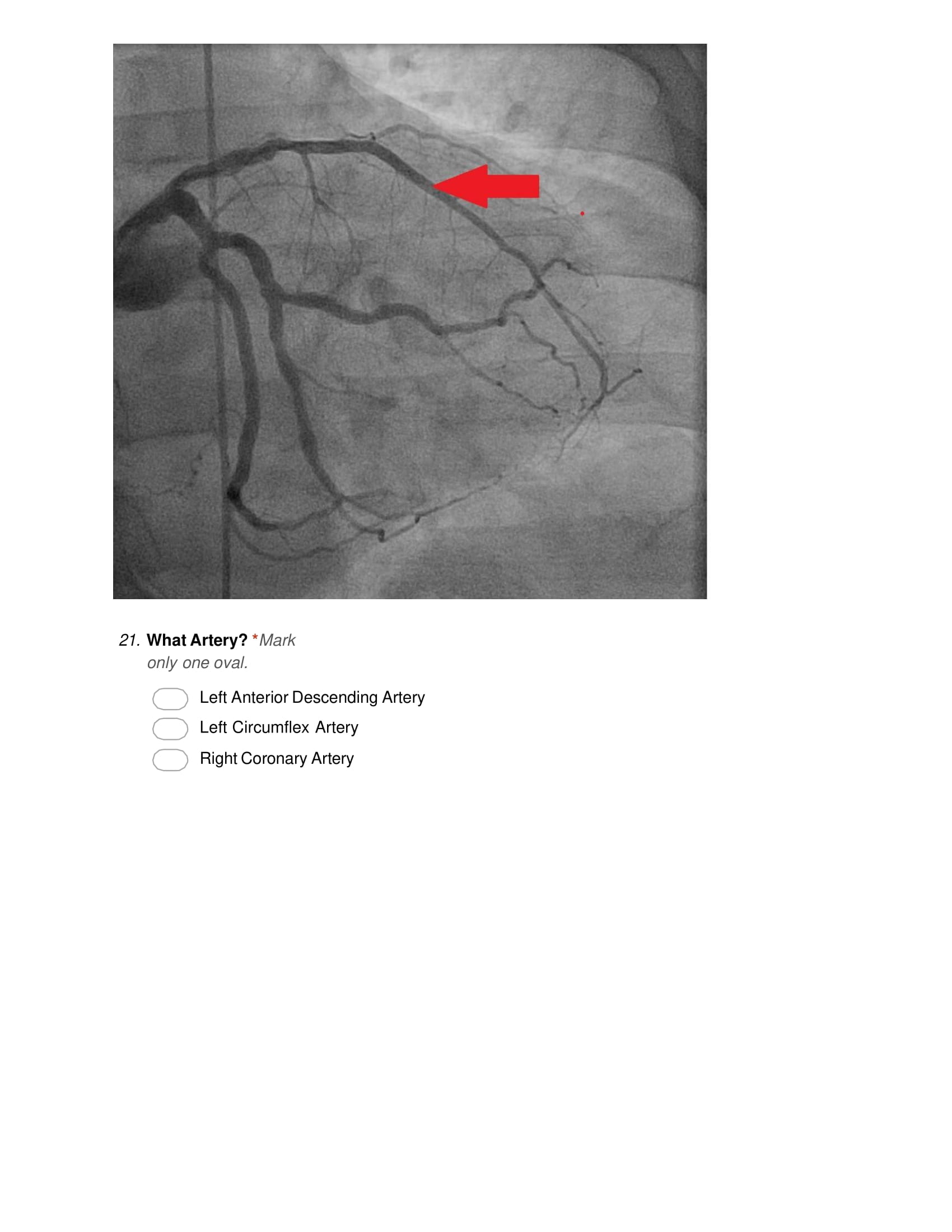

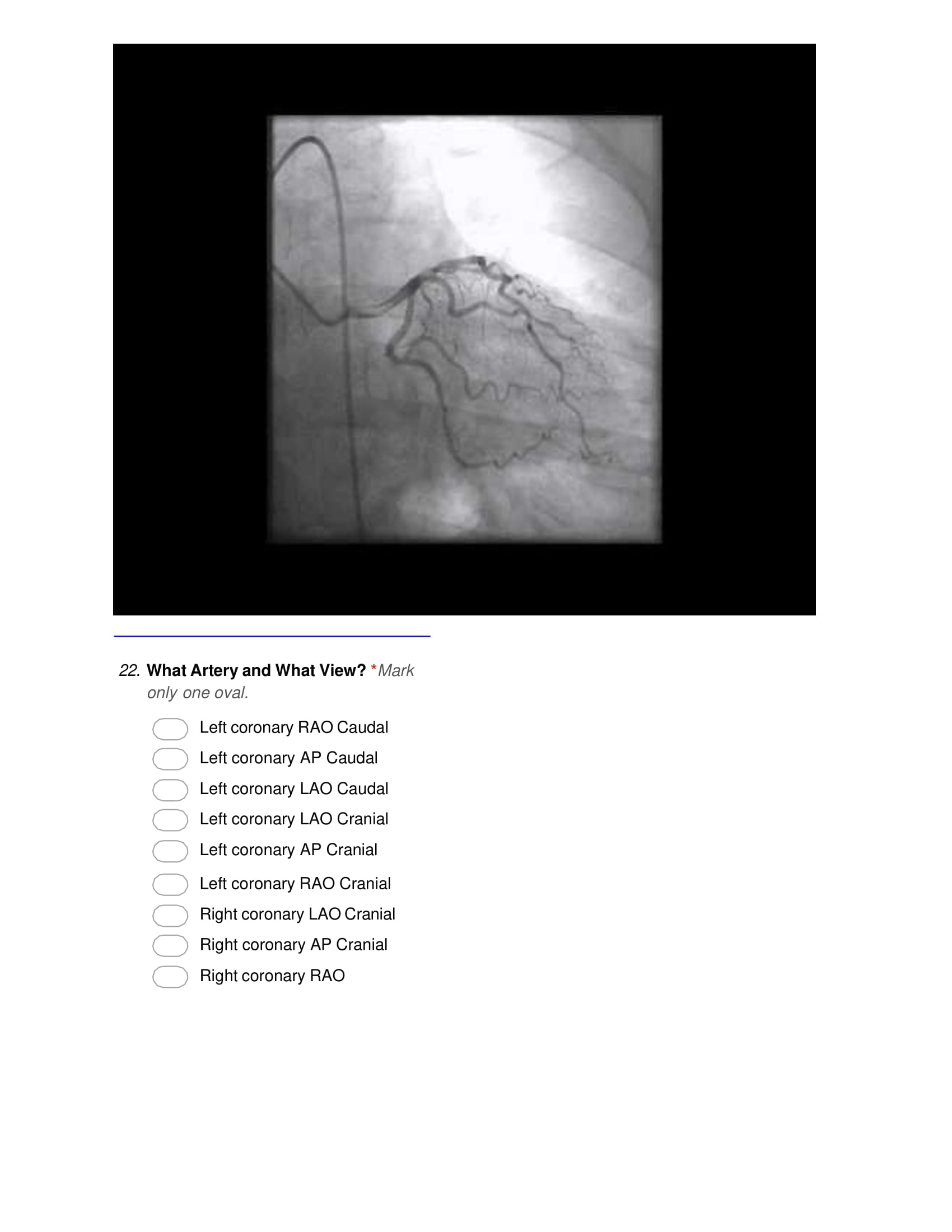

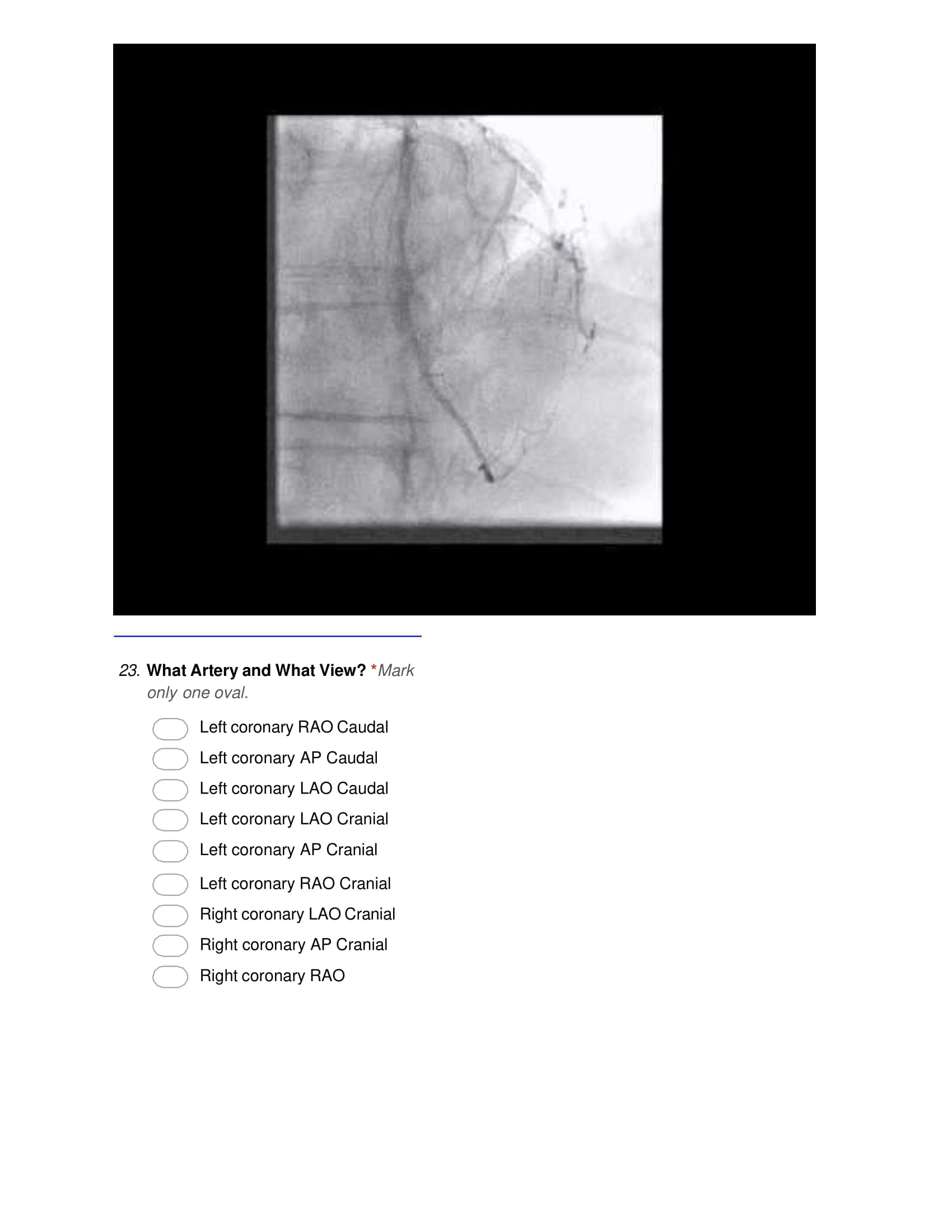

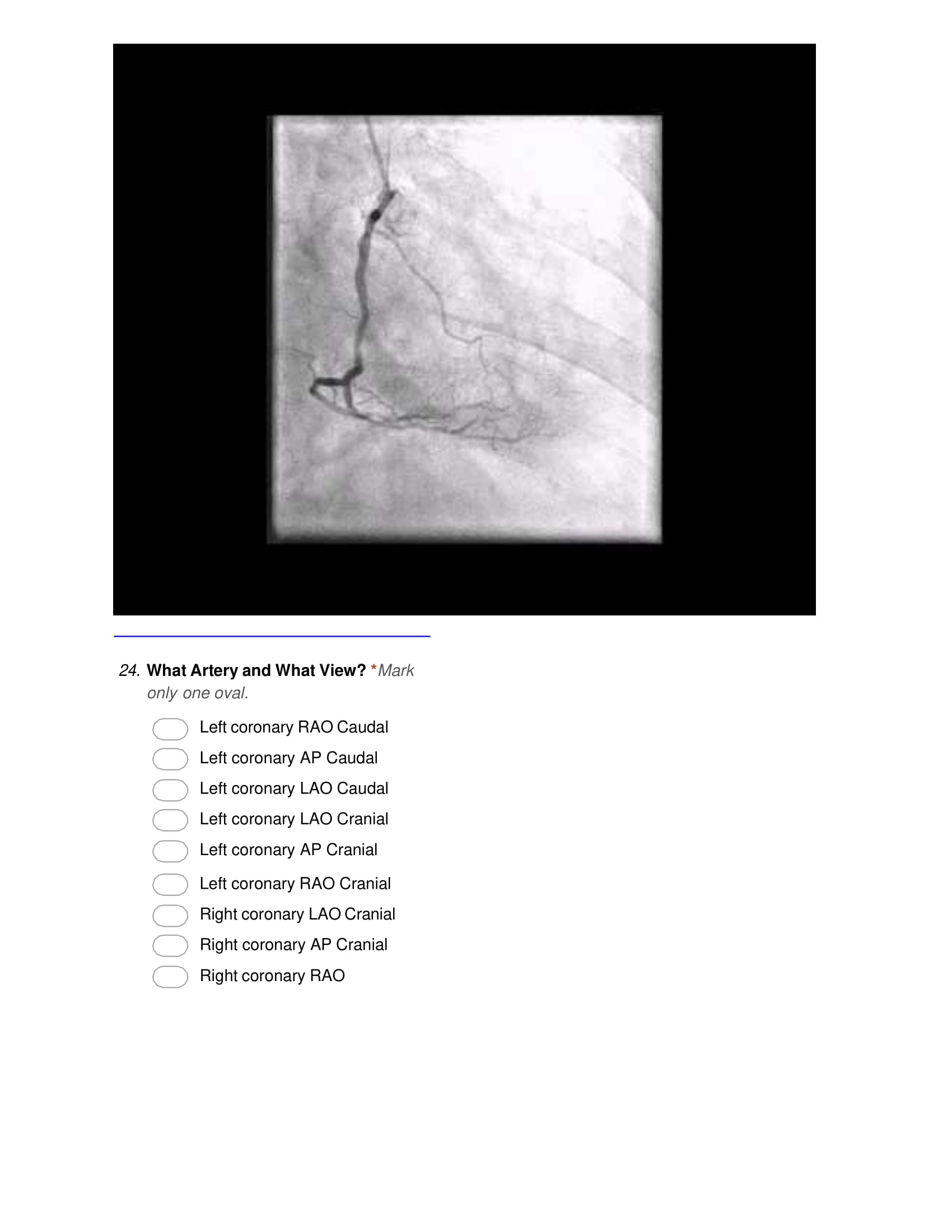

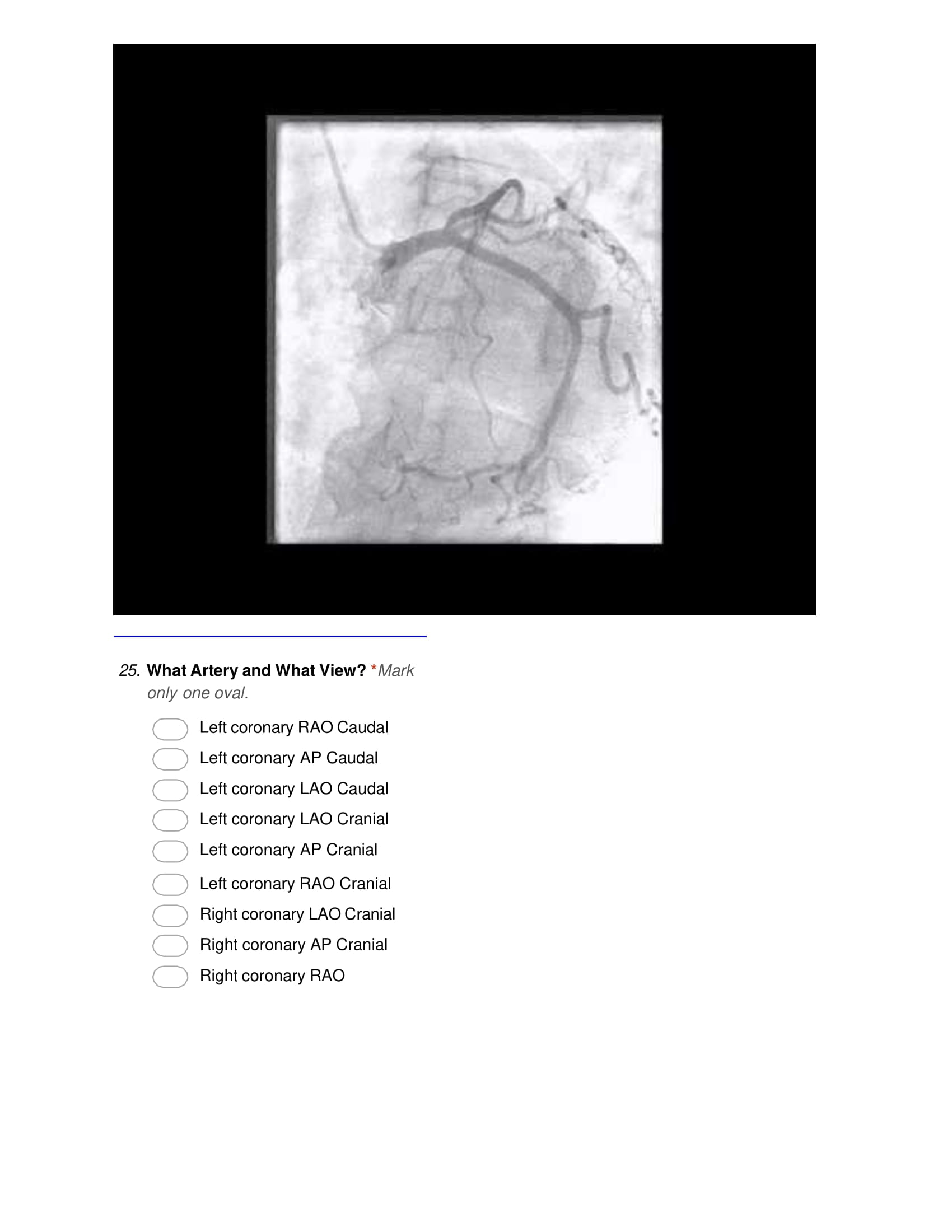

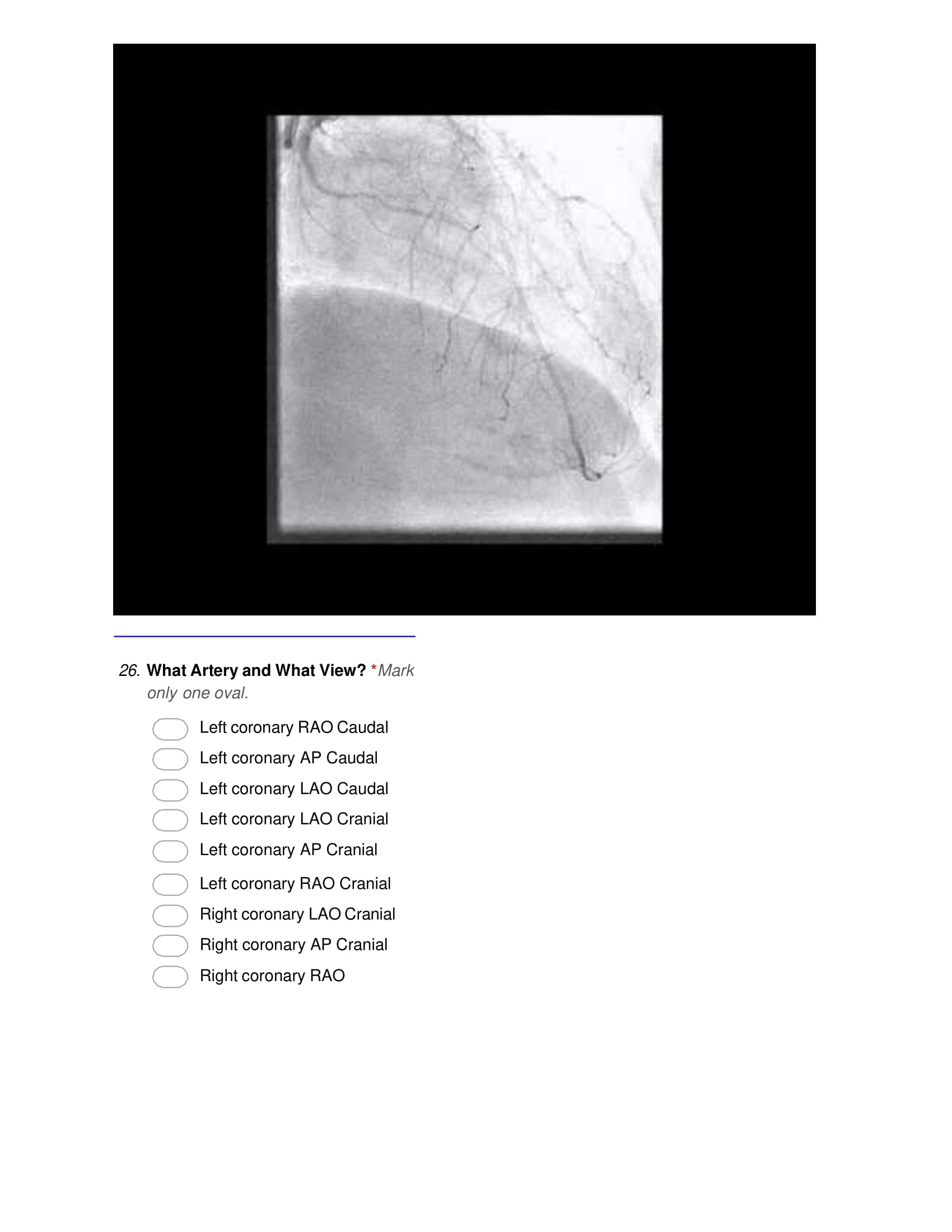

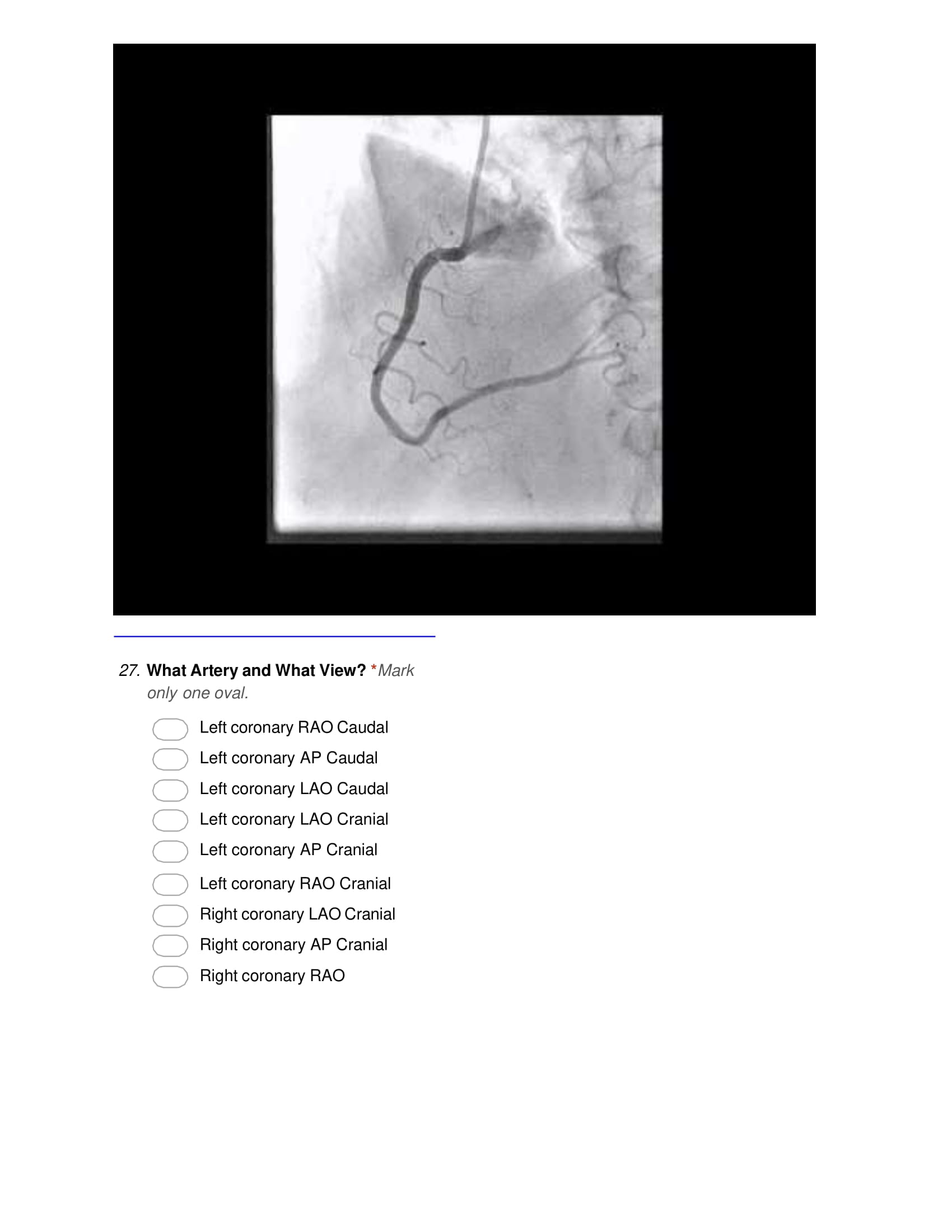

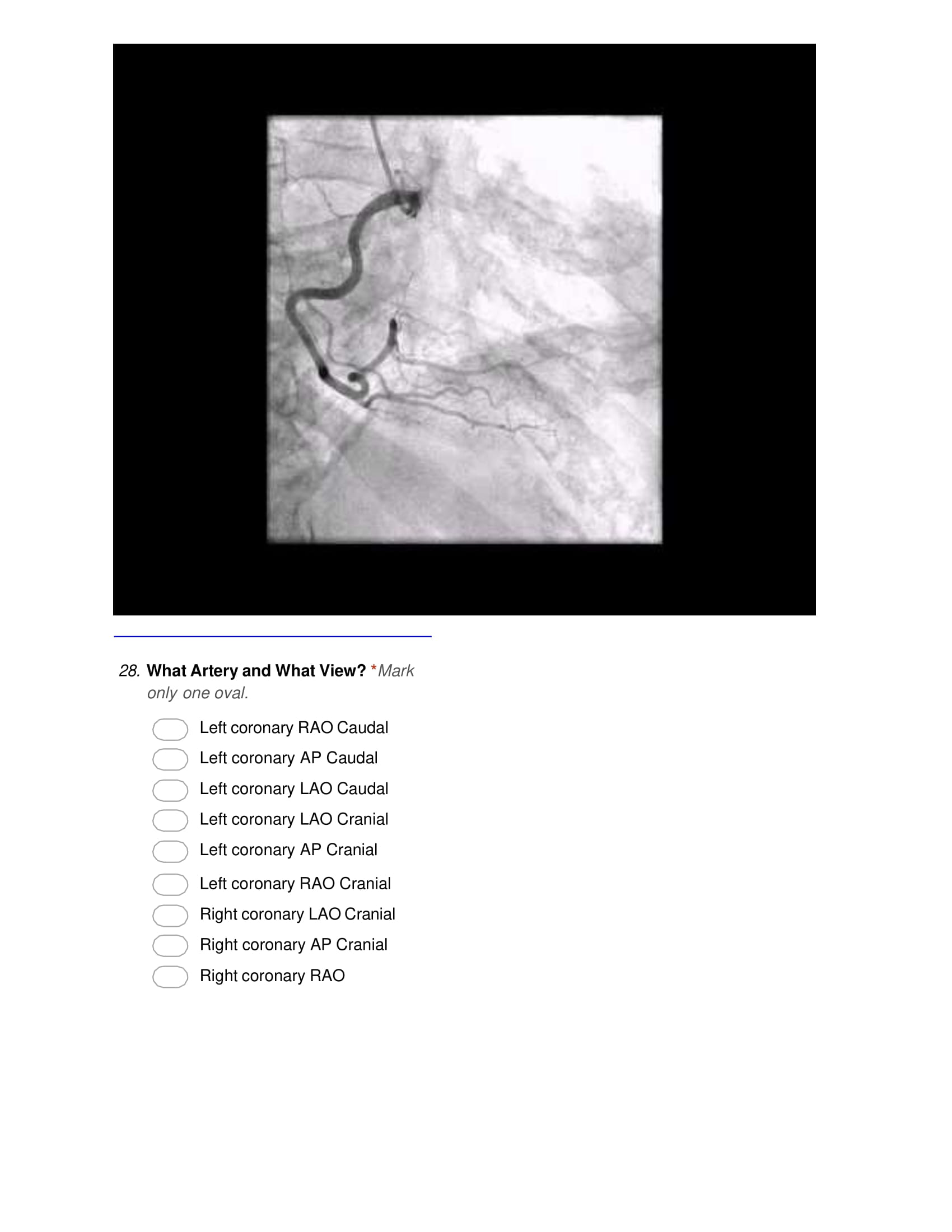

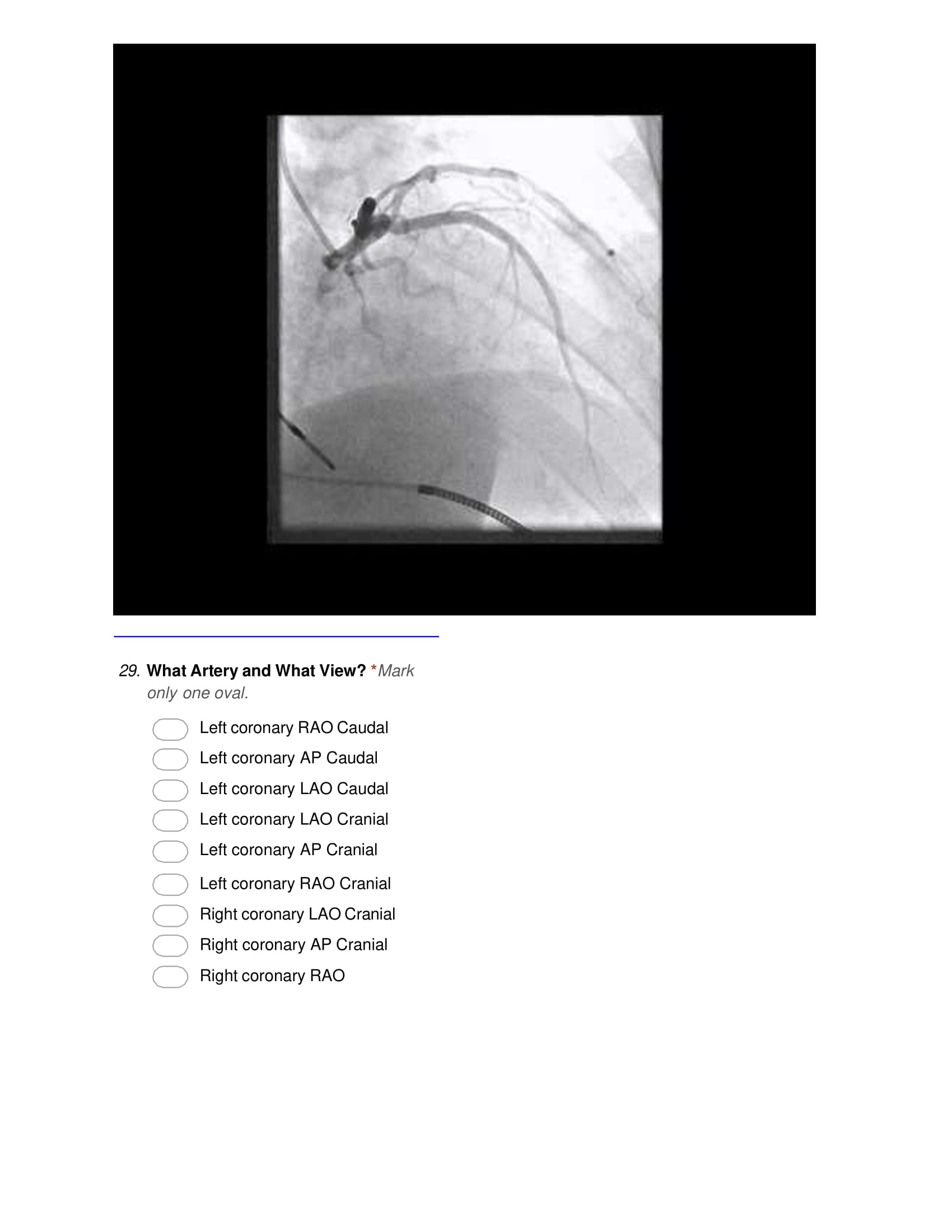

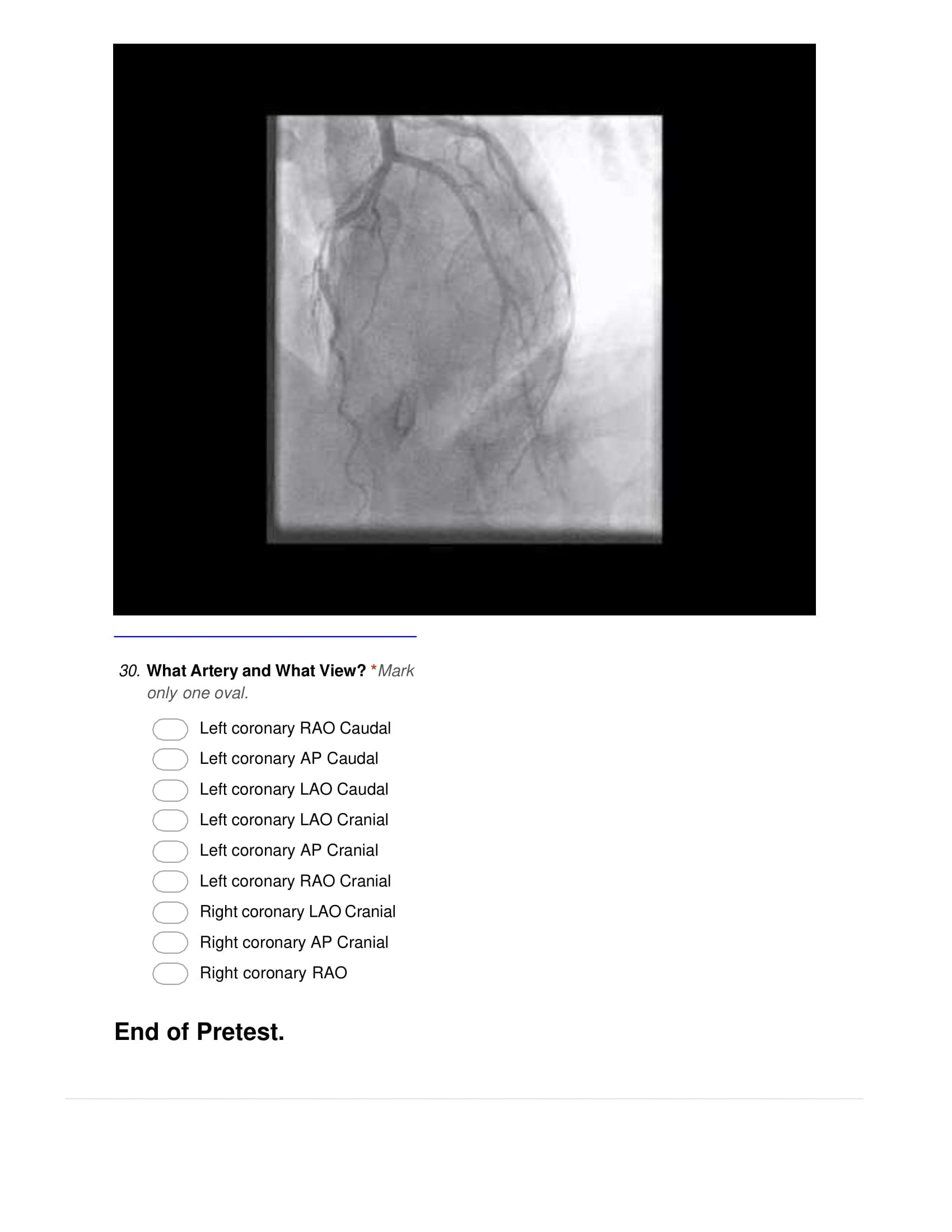


Supplementary Figure 1. Box plots demonstrating delta scores by angiographic views.


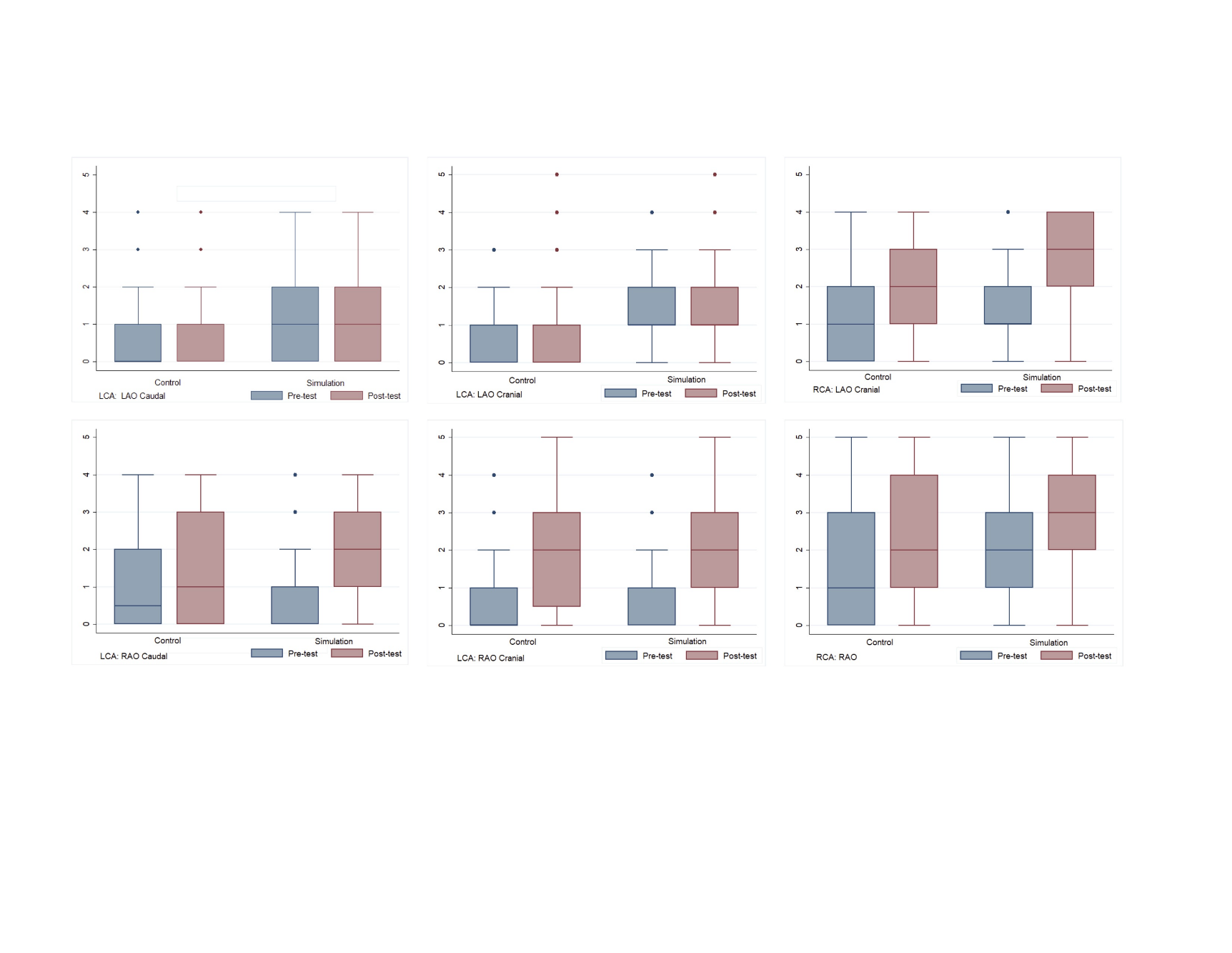

Supplement: Supplementary file 1 — Additional file 1. [file 12909_2022_3705_MOESM1_ESM.docx]
